# Boosting adsorption capacity of methylene blue dye by multiple functional ZnO-g-C_3_N_4_/ carboxymethyl chitosan/alginate –grafted polyacrylic acid composite

**DOI:** 10.1038/s41598-025-29715-7

**Published:** 2025-12-10

**Authors:** Abeer S. A. Khalaf-Allah, Maha Sultan, Yasser K. Abdel-Monem, Sabreen M. El-Gamasy, Wael A. El-Sayed, Ahmed M. Youssef

**Affiliations:** 1 Chemistry Department, Faculty of Science, El-Menuofia University, Shebin El-Kom, Egypt; 2 Packaging Materials Department, National Research Centre, 33 El Bohouth St. (former El Tahrir St.), Dokki, P.O. 12622, Giza, Egypt; 3 Photochemistry Department, National Research Centre, Chemical Industry Research Institute, Dokki, Giza, Egypt

**Keywords:** Graphitic carbon nitride, Adsorption, Dye removal, Polymer composite, Water treatment, Chemistry, Environmental sciences, Materials science

## Abstract

The present study has focused on the development of multiple functional (carboxymethyl chitosan/alginate)-grafted polyacrylic acid composite (CMCH/ALG)-g-PAA with graphitic carbon nitride (ZnO-g-C_3_N_4_) as a high-potential filler in order to satisfy the increasing demands of recycling and separation problem of ZnO-g-C_3_N_4_, which is powdery sorbent and hard to recycle after adsorption and represents a secondary pollution with high-performance in addition to strong π-π stacking interactions between g-C_3_N_4_ nanosheets can lead to serious agglomeration and restacking, which reduces g-C_3_N_4_ adsorption activity. ZnO-g-C_3_N_4_ nanosheets has been generated by traditional thermal condensation method and investigated by TEM, SEM, XRD, and FTIR. The investigation of ZnO-g-C_3_N_4_@ (CMCH/ALG)-g-PAA was done using FTIR, XRD, SEM, and TG. The highest swelling ratios of (CMCH/ALG)-g-PAA were 1697.10% at pH 8.0 and 140.5% in saline solution. Vigot and first order models fit the swelling capacity data. For (CMCH/ALG)-g-PAA with 3% ZnO-g-C_3_N_4_, the maximum adsorption capacity was 24.30 mg/g at adsorbate concentration (0.1 g/L), MB dye concentration (25 mg/L), and pH 8.0. The practical data of adsorption was well being fitted by the Langmuir, Redlich-Peterson, and Sips models indicating complicated adsorption process. Since the Δ*G*◦ values are more than − 20 kJ/mol, the adsorption is physisorption. ZnO-g-C_3_N_4_@ (CMCH/ALG)-g-PAA succeeded in effective dye removal from wastewater.

## Introduction

The safety of the world’s water supply has become a more significant issue in recent decades. As it is well known, adsorption is one of the most often used methods for getting rid of pollutants due to its low cost, simplicity of use, and other benefits^1–4^. Innovative materials with electrical, electronic, and porous properties as well as physicochemical stability have garnered a lot of interest for use in water treatment, such as graphene, carbon nanotubes, carbon quantum dots, and g-C_3_N_4_ nanosheets^5,6^. A non-metal and non-toxic polymer semiconductor, graphitized carbon nitride (g-C_3_N_4_) is created by easy thermal condensation using precursors rich in nitrogen^7,8^. G-C_3_N_4_ has several advantages over.

Graphitic carbon nitride, one of the carbon nitride allotropes has a graphene-like layered structure constructed from heptazine units and the bridge amino groups. Because of their potential uses in the adsorption of dyes and pollutants, 2D g-C_3_N_4_ nanosheets have attracted a lot of scientific attention^9,10^. Graphitic carbon nitride has a great potential to adsorb cationic dyes due to presence of abundant hydrophilic functional groups.

Graphite-like carbon nitride (g-C_3_N_4_) is a metal-free semiconductor that can be produced by simply heating urea, melamine, or cyanamide, in contrast to metal compound photocatalyst. Furthermore, the inexpensive g-C_3_N_4_ semiconductor is stable in solutions with pH 0–14 under light irradiation and has a band gap of 2.70 eV, which allows it to absorb visible light. The metal-free g-C_3_N_4_ has a lot of potential in the realm of photocatalysis because of these exceptional capabilities. However, one of the main factors limiting g-C_3_N_4_’s photocatalytic performance is the high rate of e−–h + pair recombination. In comparison to bulk g-C_3_N_4_ photocatalysts, doping with inorganic materials such as TiO_2_^11^, Zn_2_GeO_4_^12^, and others with an appropriate band potential demonstrated a higher adsorption capacity and a lower recombination rate of photogenerated charge carriers. This is a practical way to reduce the recombination rate and increase the photocatalytic and adsorption activities^13^.

Strong π-π stacking interactions between graphitic carbon nitride nanosheets, however, can cause significant restacking and agglomeration, which lowers the adsorption activity of g-C_3_N_4_^14^. Nevertheless, a number of barriers and restrictions continue to prevent g-C_3_N_4_ from being utilized in real-world applications. These include its low specific surface area, lack of active sites, and adsorption capacity, as well as the significant aggregation that occurs during the photocatalytic and adsorption processes of traditional bulk g-C_3_N_4_ generated by direct polycondensation of nitrogen-rich precursors. ZnO-g-C_3_N_4_, a powdery sorbent that is difficult to recycle after adsorption and constitutes a secondary pollutant, is another issue with recycling and separation.

To overcome these limitations, numerous attempts have been made to enhance g-C_3_N_4_’s photocatalytic capability. These include creating heterostructure, doping with heteroatoms^15^, building heterostructure^16^, synthesizing copolymers, and thermal etching^17^, among other methods. However, the easy and environmentally friendly preparation of a highly active g-C_3_N_4_ material is still preferred. Therefore, efforts have been undertaken to create g-C_3_N_4_ composites with strong hydrophilicity and dispersion for sustainable adsorption performance via situ polymerization, for instance. The adsorption and dispersion capability of g-C_3_N_4_ may be improved by chemical modification of its surface functional groups, which serve as chemical binding sites^18^. Similarly, due to structure similarity between graphene and graphitic carbon nitride and to avoid π-π stacking, a new reduced graphene oxide was added to the gum tragacanth-cl-N, Ndimethylacrylamide (GT-cl-poly(DMA)/RGO) hydrogel composite. The reported maximum adsorption of Hg2 + and Cr6 + by (GT-cl-poly(DMA)/RGO) hydrogel composite was 666.6 mg g^− 1^ and 473.9 mg ^g−1^, respectively and was fitted with Langmuir and pseudo-second-order isotherms^19^. Also, Sahraei et al. (2016) reported adsorption of Cr^6+^ metal by chitosan/reduced-graphene oxide/montmorillonite composite hydrogel with maximum adsorption of 87.03 mg g^− 1^^20^. Polyaniline (PANI) chains were embedded at the margins of graphene oxide nanosheets via in situ chemical oxidation polymerization. The maximal adsorption of brilliant green was 142.8 mg/g at pH 7 and well fitted with pseudo-second-order kinetic models and the Langmuir isotherm^18^. Sodium carboxymethylcellulose (CMC-Na)/polyvinyl alcohol (PVA) hydrogel reinforced with cellulose nanocrystal (CNC) and graphitic-like carbon nitride (g-C_3_N_4_) was fabricated. Methylene blue’s adsorption capability (MB, qe > 198.6 mg/g). The Langmuir isotherm model and the pseudo-second-order kinetic model both fit the adsorption characteristics of MB on the hydrogel as it was manufactured. It was demonstrated that the high adsorption capacity of the hydrogels was caused by the interplay of hydrogen bonds and π-π stacking^21^. Polyacrylic acid-grafted hydroxyethyl cellulose and polyvinyl alcohol that are physically cross-linked, (PAA-g-(HEC-PVA)- Fe^3+^ / ZnO-g-C_3_N_4_, was created to increase the adsorption capacity of methylene blue dye (MB). The optimal adsorption which fit the pseudo-second-order kinetic Langmuir adsorption model was 216.82 mg/g up to 50 min^22^.

Ethylenediaminetetraacetic acid (EDTA) functionalized graphene oxide-chitosan nanocomposite (GO-EDTA-CS) was synthesized by Monu Verma et al. in 2022. The adsorption of MB was studied using the Langmuir isotherm model, with a maximum adsorption capacity of 141 ± 6.60 and mg g^− 1^ 15 at solution pH of 5.10 and 8.30, respectively, starting concentrations of 100 mg L^− 1^ for MB dye, at 240 min of contact time and an adsorbent dose of 50 mg (in 40 mL solution)^23^. Graphene oxide (GO) and ethylenediaminetetraacetic acid (EDTA) have been used to functionalize chitosan (CS) in order to remove organic pollutants from wastewater, such as sildenafil (SDF) and ciprofloxacin (CIP). At contact time 120–180 min, pH 5–8, and 25 mg of adsorbent in 20 mL solution, the produced adsorbent demonstrated heterogeneous adsorption capacities of 75.40 and 40.90 mg g^− 1^ for CIP and SDF, respectively^24^. In order to remove organic contaminants simultaneously, a trifunctional β-cyclodextrin-EDTA-chitosan polymer adsorbent was also created utilizing a straightforward chemical process that involved crosslinking via EDTA. For MB dye, the adsorbent’s highest adsorption capacities were 107.20 ± 5.70. A novel graphene oxide-doped β-cyclodextrin chitosan polymer^25^ and an ethylenediaminetetraacetic acid-functionalized graphene oxide-chitosan nanocomposite shown high adsorption capabilities of up to 158.40 mgg^− 1^ and 121 mgg^− 1^ for MB, respectively ethylenediaminetetraacetic acid functionalized graphene oxide-chitosan nanocomposite^23^.

Many attempts have been undertaken to combine with producing copolymers to develop g-C_3_N_4_-based materials with strong adsorption^26^. Adsorption is regarded as an appropriate method since it can eliminate water contaminants like organic dyes and heavy metals entirely, even from diluted solutions, generates non-toxic byproducts, is affordable, permits the recycling of biosorbents, and helps recovery^27^. Many studies deal with utilizing adsorbents modified with graphitic carbon nitride, for example, polyacrylonitrile (PAN) support impregnated with amine-functionalized graphitic carbon nitride/magnetite (g-C_3_N_4_-NH_2_/Fe_3_O_4_) composite nanofibers (PAN/gC_3_N_4_-NH_2_/Fe_3_O_4_) was used to create a very stable mixed-matrix adsorbent system using simple electrospinning processes. According to the experimental results of the batch investigation, 0.02 g of adsorbent dose could adsorbed 97.0 and 99.0% of arsenite (As(III)) and arsenate (As(V)), respectively, within 60 min of contact time at pH 7 and 4, with an initial concentration of 10 mg/L^28^. For the efficient static and dynamic adsorption of Pb^2+^ from aqueous media, Reem Ghubayra (2025) created a new graphitic carbon nitride/chitosan composite modified with thiosemicarbazide (TGCS). TGCS showed that at an adsorbent dosage of 1.5 g/L, pH 5, 45 min of shaking, and 23 °C, the Langmuir adsorption capacity for Pb^2+^ was 329.61 mg/g^29^. The porous graphitic carbon nitride surface (g-C_3_N_4_) was modified with 2-amino fluorene polymer (AFP) to build a new hybrid adsorbent (g-C_3_N_4_/AFP). Its adsorptive activity was surveyed for the removal of heavy metals, methylene blue, and malachite green dyes from aqueous solution. According to the results, the maximal adsorption capacities of g-C_3_N_4_ and g-C_3_N_4_/AFP were 184.51 and 452.19 mg g^− 1^ for Cu (II), 85.73 and 221.85 for methylene blue, and 226.88 and 327.83 for malachite green^30^.

Similarly, the composite membrane modified with graphitic carbon nitride is promising performance option and potential for scale-up at the industrial level^31^. The graphitic carbon nitride (g-C_3_N_4_) nanocomposite polyethylene terephthalate (PET) micro plastic membranes were fabricated using the phase inversion technique by immersing g-C_3_N_4_ nanoparticles into the PET casting solution. This membrane could remove 57.6% and 51.2% of organic matter when using pristine PET membrane for drinking water and wastewater effluent, respectively. Whereas, with PET/g-C_3_N_4_ membrane, removed up to 72.8% and 72.3% for drinking water and wastewater effluent, respectively. In addition, the composite membrane demonstrated good stability and reusability for up to 8 filtration cycles^32^. Hydrogels, a significant family of polymeric materials distinguished by their soft appearance, shape permanence, and swelling characteristics, are formed by polysaccharides^33–36^. Because hydrogels make it easier for molecules to diffuse into the gel network, polysaccharides can be used in a variety of ways. The immobilization of g-C_3_N_4_ on hydrogels, polysaccharides, and hydrogel-based composites for water pollutants adsorption is the subject of relatively few investigations^37–39^.

The graphitic carbon nitride composite hydrogels (g-C_3_N_4_/SA) modified by alginate were effectively prepared through a simple cross-linking polymerization technique, sodium alginate is effectively used to functionalize the graphitic carbon nitride and create SA modified g-C_3_N_4_ (also known as g-C_3_N_4_/SA). Using epichlorohydrin as a crosslinker and ammonium persulfate (APS) as an initiator, polyacrylamide was produced by polymerizing acrylamide, which exhibited good flocculation. Pb(II), Ni(II), and Cu(II) had maximal adsorption capacities of 383.4, 306.3, and 168.2 mg g^− 1^, respectively^40^. Huiqiang Wang et al. (2020) have created as a smart sodium carboxymethylcellulose (CMC-Na)/polyvinyl alcohol (PVA) hydrogel reinforced with cellulose nanocrystals (CNC) and graphitic-like carbon nitride (g-C_3_N_4_) with proper mechanical, thermal, and swelling response. Outstanding tensile strength (up to 648 KPa), excellent elongation (1169%), and satisfactory toughness (340 KJ/m3) were all exhibited by the 1.0% g-C_3_N_4_/CNCs-H hydrogel.The g-C_3_N_4_/CNCs-H hydrogel as synthesized has a high equilibrium swelling capacity (90.47–117.3 g/g), which significantly increased the methylene blue adsorption capacity (MB, Q_e_ > 198.6 mg/g)^21^. Zhongyue Chen et al. (2022) used a simple sol-gel technique to develop environmentally friendly sugarcane cellulose (SBC)/sodium carboxymethylcellulose (CMC-Na) adsorbent augmented with carbon nitride (g-C_3_N_4_) with pseudo-second-order kinetic model and Langmuir adsorption capacity of 362.3 mg g^− 1^. The hydrogel composite showed excellent stability and reusability with virtually little adsorption capacity loss and high selectivity to MB/MO or MB/RhB mixed dyes^41^. In another investigation, a novel palladium nanocatalyst supported on graphitic carbon nitride (g-C_3_N_4_) and sodium carboxymethyl cellulose (Na-CMC) composite hydrogel beads was fabricated. This composite was applied to diminish several nitroaromatics such as 4-nitrophenol (4-NP), 2-nitrophenol (2-NA), 4-nitroaniline (4-NA), 4-nitro-*o*-phenylenediamine (4-NPDA), and organic dyes as well as methylene blue (MB), methyl orange (MO), Rhodamine B (RhB), in addition to potassium hexacyanoferrate(III) (K_3_[Fe(CN)_6_]). Even after four consecutive runs, Pd@Na-CMC/g-C3N4 demonstrated excellent stability and was able to lower 4-NP and MO without suffering a noticeable decline in performance^42^.

The key prospect of this study is to fabricate composite adsorbent based on (carboxymethyl chitosan/alginate)-grafted polyacrylic acid (CMCH/ALG)-g-PAA modified with (ZnO-g-C_3_N_4_) of multiple functionalities for high efficient methylene blue dye remediation. Initially, ZnO-g-C_3_N_4_ nanosheets was prepared by conventional thermal condensation technique and investigated by TEM, SEM, XRD and FTIR. ZnO-g-C_3_N_4_@(CMCH/ALG)-g-PAA was prepared and investigated by FTIR, XRD, and SEM. Thermal analysis of (CMCH/ALG)-g-PAA and ZnO-g-C_3_N_4_@(CMCH/ALG)-g-PAA was investigated. The swelling behavior of CMCH/ALG)-g-PAA were investigated and their kinetics were investigated at pH 5.8, 7.0, 8.0 and saline solution. The adsorption optimization including MB, adsorbent, adsorbate concentrations, and pH were explored. The isotherms, adsorption mechanism was proposed, and thermodynamics studies were explored.

## Materials and methods

### Materials

Acrylic acid (AA) of Molecular weight (72.06 g/mole) was purchased from Sigma Aldrich, carboxymethyl chitosan of 90% deacetylation degree and 0.9 of substitution degree was delivered from Macklin Company (Shanghai, China), alginate sodium salt (ALG), Potassium persulfate (PPS), methylenebisacryalmide (MBA) were delivered from Merck. Analytical grade (AR) zinc acetate dihydrate, melamine, urea, sodium hydroxide, and hydrochloric acid were purchased from Loba chemicals. Methylene blue (MB) dye was purchased from TCI chemicals and employed as a model water pollutant in the current study.

### Synthesis of graphitic carbon nitride (g-C_3_N_4_) nanosheets

G-C_3_N_4_ was prepared using the traditional method of thermal condensation. Briefly, 5 g of melamine was initially pounded well into fine powder with pestle and mortar for 30 min and dried in a furnace at 105 °C. The dry powder was then placed in a silica crucible, covered with aluminum foil, and calcined for six hours at 600 °C in a furnace. g-C_3_N_4_ was produced as a pale yellow powder^43^. The g-C_3_N_4_ product powder was dispersed in deionized water and ultrasonically pulverized for two hours to create ultrathin porous nanosheets. For additional investigation, the solid nanoparticles were subsequently collected using a centrifuge and dried for 24 h at 60 °C for further analysis^44^.

### Synthesis of zinc oxide -graphitic carbon nitride (ZnO- g-C_3_N_4_) hybrid nanosheets

ZnO-graphitic carbon nitride (ZnO-g-C_3_N_4_) has been synthesized using the traditional thermal condensation method. As precursors, zinc acetate dihydrate and melamine were thoroughly combined in a 4:1 weight ratio. A specific quantity of the thoroughly combined zinc acetate and melamine was put in a furnace and heated for six hours at 600 °C at a rate of 2 °C per minute. The product was then cleaned with a lot of deionized water and allowed to dry for 24 h at 60 °C^43^. Ultrasonic pulverization was used for two hours to exfoliate the product powder in deionized water. After that, the solid nanoparticles were gathered using a centrifuge and dried for 24 h at 60 °C for additional examination.

### Synthesis of (carboxymethyl chitosan/alginate)-grafted polyacrylic acid (CMCH/ALG)-g-PAA

**Scheme 1:**
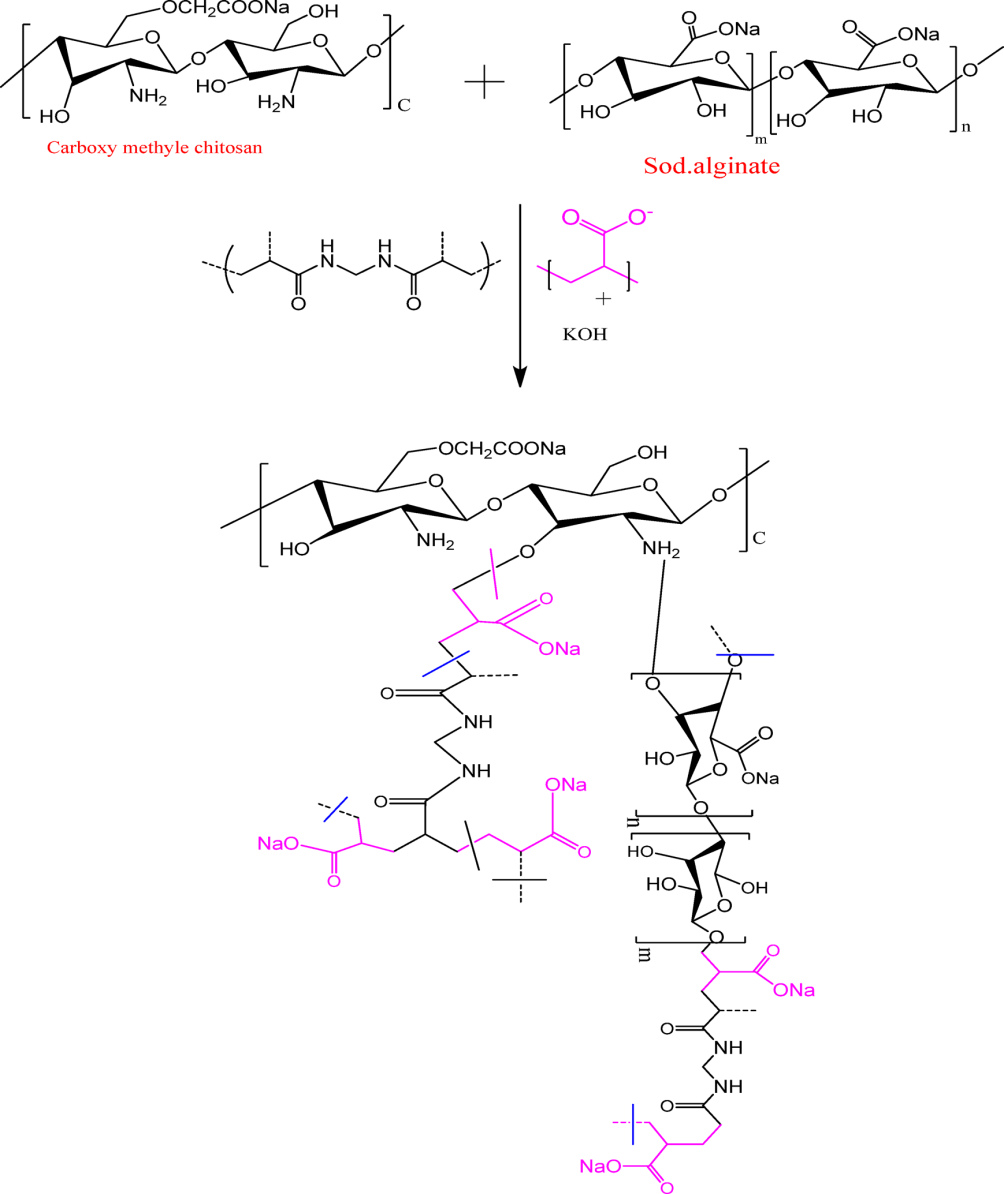
Proposed grafting mechanism of (CMCH/ALG) polymer blend with acrylic acid chemical structure.

First, 10 ml of deionized water in a 100 ml beaker was used to completely dissolve the MBA cross-linker (0.05 g) by stirring. Next, 0.32 g of a 1:1 weight ratio CMCH/ALG combination was added. After being neutralized with a sodium hydroxide solution (5*M*), 5.25 mL of AA was added to the beaker and constantly swirled. Potassium persulfate (0.05 g/10 mL), the redox initiation system, was then added to the beaker, agitated for two minutes, and then placed in a water bath set at a constant temperature (70 °C) for four hours. The reaction product was oven-dried after being thoroughly cleaned with deionized water (3 × 500 ml, 24 h)^45^. The nanocomposite was dried in an oven at 60 °C after the homopolymer was removed from (CMCH/ALG)-g-PAA by solvent extraction with acetone (Scheme 1).

### Synthesis of ZnO-g-C_3_N_4_@(carboxymethyl chitosan/alginate)-grafted polyacrylic acid

**Scheme 2:**
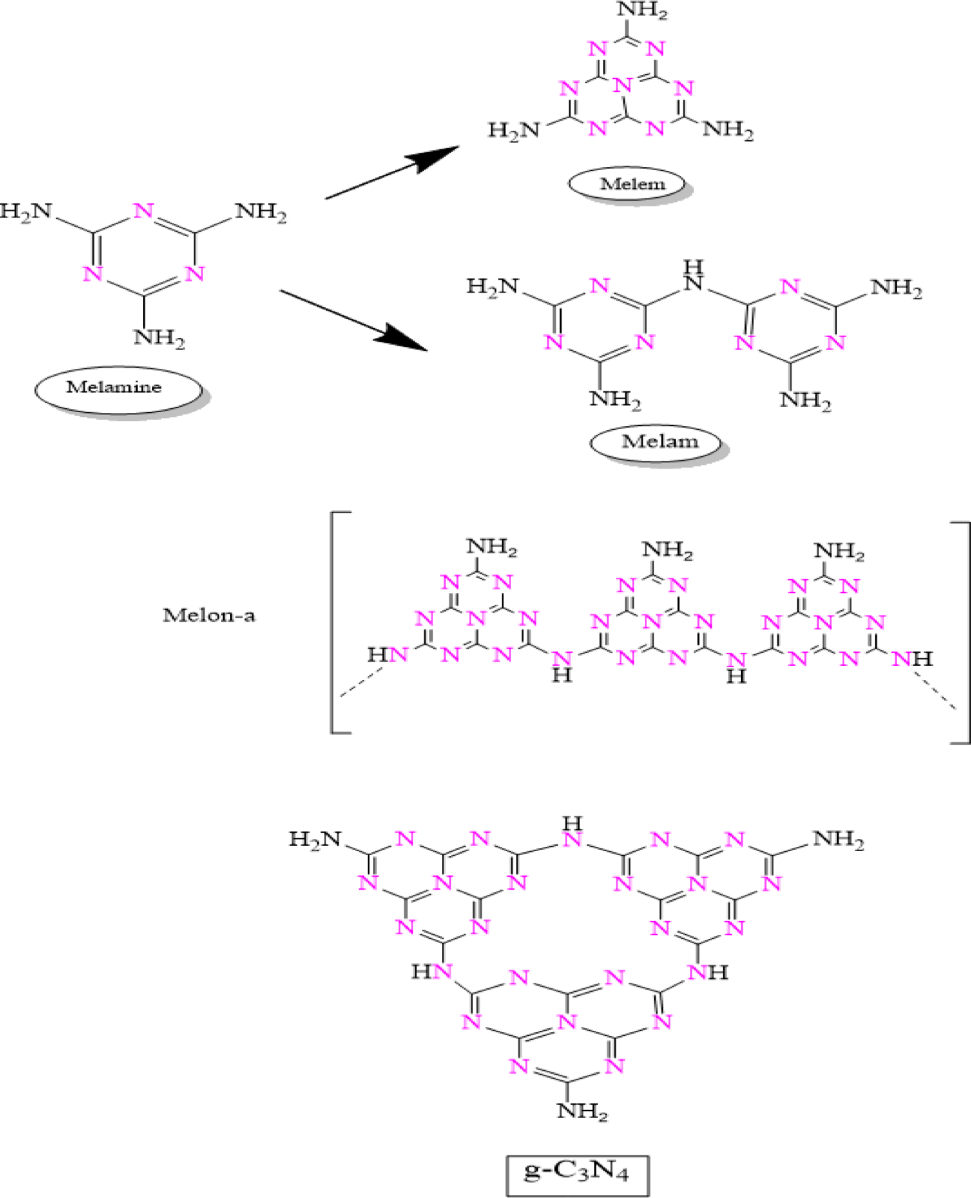
Illustrate melamine and its thermal condensation intermediate products and g-C_3_N_4_ chemical structures.

First, 10 ml of deionized water in a 100 ml beaker was used to completely dissolve the MBA cross-linker (0.05 g) by stirring. Next, 0.32 g of a 1:1 weight ratio CMCH/ALG combination was added. After neutralizing the sodium hydroxide solution (5 mol/l, 2.04 ml), AA (5.25 mL) was added to the beaker and constantly swirled. ZnO-g-C_3_N_4_ was added as 1%, 2%, and 3% based on total weight of polymer formulation. The mixture was then placed in a water bath with a constant temperature of 70 °C for four hours after the redox initiation system, PPS (0.05 g/mL), was added to the beaker and swirled for two minutes. After being thoroughly cleaned with deionized water (3 × 500 ml, 24 h), the reaction product was oven-dried^45^ (Scheme 2).

### Characterization

#### Attenuated total reflectance infrared spectroscopy [ATR-IR] analysis

Fourier Transformation Infrared (FTIR) spectra were obtained at room temperature using the Attenuated Total Reflection (ATR) unit attached with FTIR-Vertex 70 Bruker, Germany, in the range of 4000 –400 cm^− 1^, to distinguish the chemical compositions of g-C_3_N_4_, ZnO-g-C_3_N_4_ nanosheets, (CMCH/ALG)-g-PAA, and ZnO-g-C_3_N_4_@(CMCH/ALG)-g-PAA copolymers.

#### X-ray diffraction analysis (XRD)

Using a Bruker diffractometer, g-C_3_N_4_, ZnO-g-C_3_N_4_ nanosheets, (CMCH/ALG)-g-PAA, and ZnO-g-C_3_N_4_@(CMCH/ALG)-g-PAA copolymers diffractions were examined (Bruker D 8 advance target). It was done using a 40 kV, 40 mA CuK radiation source with a second monochromator (λ = 1.5405 Ǻ). The scanning rate for line broadening profile analysis and phase identification was 0.2 min^− 1^.

Equation (1) was used to measure the crystallinity indices (*CI* %) of the samples.

1 $$CI~\% ~ = \frac{{A_{C} }}{{A_{C} + A_{a} }} \times 100$$

where, *A*_c_ and *A*_a_ are crystalline area and amorphous, respectively^46^.

#### Transmission electron microscopy (TEM) analysis

JEM-10OCXII TEM (Japan) operating at 120 KV was used to analyze g-C_3_N_4_ and ZnO-g-C_3_N_4_ nanosheets using transmission electron microscopy (TEM). To create a very thin film, the freshly prepared specimen solutions were dropped on the copper grid that had been coated with carbon. The sample was ready to be examined after 15 min.

#### Surface morphology studies (SEM&EDS)

The surface morphologies of g-C_3_N_4_, ZnO-g-C_3_N_4_ nanosheets, (CMCH/ALG)-g-PAA, and ZnO-g-C_3_N_4_@(CMCH/ALG)-g-PAA copolymers were explored using SEM Model Quanta 250 FEG (Field Emission Gun) attached with EDX Unit (Energy Dispersive X-ray Analyses), with accelerating voltage 30 K.V., magnification14x up to 1000000 and resolution for Gun.1n).

#### Swelling ratio percentage (Sr %)

The swelling ratios of (CMCH/ALG)-g-PAA copolymers were described in details as a function of time change with the use of buffer solution of different pH 5.8, pH 7.0, and pH 8.0 and saline solution (5% NaOH). To calculate the swelling ratio %, (Eq. 2) was employed.

2 $$\:Sr\:\text{\%}=\frac{\left({\text{W}}_{\text{t}}-{\text{W}}_{\text{o}}\right)}{{\text{W}}_{\text{o}}}\:\times\:100$$

where *W*_t_ is the sample’s weight at time (*t*), and *W*_o_ is the sample’s initial weight.

##### Swelling kinetics

The swelling dynamics using a pseudo- first and second order formula was utilized. The rate at the time (*t*) was inserted into Eqs. 3 and 4, respectively.

3 $$\:\text{Sr}\text{\:t},\:f=\:{\text{Sr}}_{\text{e}},f\:\left(1-{e}^{-{\text{k}}_{\text{1,\:f\:\:}}t\:}\right)$$

4 $$\:\text{Sr}\text{\:t,\:s}=\frac{{{\text{\:Sr}}_{\text{\:e}}}^{2}{\text{k}}_{\text{2}},s\:t}{{{\text{Sr}}_{\text{e,\:S\:}}\text{k}}_{\text{2}},s\:t+1}$$

where $\:\text{Srt},\:f$ and $\:\text{Srt}\text{,\:s}$ are the sample`s swelling ratio at time t, $\:{\text{Sr}}_{\text{e}},f$ and $\:{\text{Sr}}_{\text{e,\:S\:}}$ are the sample`s swelling ratio at equilibrium and $\:{\text{k}}_{\text{1,\:}\text{f\:}}$ and $\:{\text{k}}_{\text{2}},s$ are the rate constant of first and second order reactions.

Next, using Eq. (5), the swelling values derived from the data are fitted into a Voigt model.

5 $$\:{\text{Sr}\text{\:t}}_{\text{,\:\:V}}=\:{\text{Sr}}_{\text{e}},\:V\:\left(1-{e}^{-t/r,V}\right)$$

Where *St*,_,V_ is the swelling capacity at time *t*. The swelling capacity at infinite time, or the greatest water-holding capacity, is known as equilibrium swelling (*St*_e, V_), and *r*,V is the rate parameter, which is the time required to reach 0.63 of the equilibrium swelling^47,48^.

Chi-square ($\:{\upchi\:}2$) was used to measure the precision of the test findings (Eq. 6)

6 $$\:\text{C}\text{h}\text{i}-\text{S}\text{q}\text{u}\text{a}\text{r}\text{e}\:\text{s}\text{t}\text{a}\text{t}\text{i}\text{s}\text{t}\text{i}\text{c}\text{s}\:\left({\upchi\:}2\right)=\:\sum\:\frac{{(Sr}_{e}{,\:}_{\text{e}\text{x}\text{p}}-{Sr}_{e,\:}{,}_{cal{)}^{2}\:\:\:}}{\:{Sr}_{e,\:}{,}_{cal}}$$.

#### Thermal properties

Universal V4.5 A TA Instruments SDT Q600 V20.9 Build 20) was utilized to carry out thermogravimetric analysis of polymer composite at a uniform heating rate of 10 °C /min in the temperature range from room temperature up to 1000 °C. A comparative estimation of the activation energies (*E*_a_) using Coat and Redfern’s (1964). Equation (7) was employed when *n* ≠ 1:

7 $$\:\text{log}\left(\frac{1-{\left(1-\alpha\:\right)}^{1-n}}{{T}^{2}\left(1-n\right)}\right)=\text{log}\frac{AR}{bEa}\left(1-\frac{2RT}{Ea}\right)-\frac{Ea}{2.3RT}$$

Where *α is* the thermally-decomposed mass (deg/min) at the time (t) and *T* is the absolute temperature and *n* from 0.0 to 3.0 with 0.5 increments.

#### Practice of dye adsorption

Batch adsorptive tests were performed in a conical 100 mL flask to evaluate several factors, such as the initial dye dosages (5 ppm, 10 ppm, 15 ppm, 20 ppm, and 25 ppm), pH (5.8, 7.0, and 8.0), and the amount of adsorbent (100, 200, and 300 mg). Sodium phosphate buffers were used in the experiments. The dye concentration was measured by measuring the absorbance value at λ_max_ = 665 nm using UV/Vis spectrophotometer (JASCO V-730, Japan). The sample’s equilibrium adsorption capacity (*Q*_e_, mg/g) and adsorption capacity (*Q*t, mg/g) at time *t* were calculated separately in Eqs. (8) and (9).

8 $$\:\text{Q\:}\text{t}=\frac{({\text{C}}_{\text{0}}-{\text{C}}_{\text{t}})\:}{m}V$$

9 $$\:{\text{Q}}_{\text{e}}=\frac{({\text{C}}_{\text{0}}-{\text{C}}_{\text{e}}\text{)}}{m}\:V$$

where *C*_0_, *C*e and *C*_t_ (mg/L) are initial concentration, concentration at equilibrium, and concentration with time ( t ) of MB (ppm). The mass concentration of the MB solution at equilibrium was *C*_e_ (mg/L). *V* is the volume of the MB solution (*L*), and *m* is dry weight of (CMCH/ALG)-g-PAA grafts and ZnO-g-C_3_N_4_@(CMCH/ALG)-g-PAA grafts (g).

##### Kinetics study

The process of adsorption was assessed using its kinetics and equilibrium, two crucial physico-chemical features. The adsorption kinetics data were analyzed using three rate equations: Lagergren pseudo-first-order (Eq. 10), Ho’s pseudo-second-order (Eq. 11), and Elovich reaction kinetics (Eq. 12)

10 $$\:{\text{Qt}}_{\text{\:,\:f}}=\:{\text{Q}}_{\text{e,\:f}}\:\left(1-{e}^{-{\text{k}}_{\text{1,\:f\:}}t\:}\right)$$

11 $$\:{\text{Qt}}_{\text{\:,\:s}}=\frac{{{\text{Q}}_{\text{e,\:s}}}^{2}{\text{k}}_{\text{2}},s\:t}{{{\text{Q}}_{\text{e,\:s}}\text{k}}_{\text{2\:,s}}t+1}$$

12 $$\:{\text{Qt}\text{,}}_{\text{\:\:v}}=\:\frac{1}{\beta\:}\:\:\:ln\:\left(1+\alpha\:\beta\:\:t\right)$$

Where *k*_1, f_ (min^− 1^) is Lagergren pseudo-first-order rate constant of the adsorption process and $\:{\text{Qt}}_{\text{,\:f}}$ is the adsorption capacity (mg/g) at time (*t*), $\:{\text{Q}}_{\text{e,}\text{\:f}}\:$ is adsorption capacity (mg/g) at equilibrium. $\:{\text{k}}_{\text{2}},s$ (g.mg^− 1^.min^[− [1^) is Ho`s pseudo-second -order rate constant $\:\alpha\:$ is the initial adsorption rate for Elovich model (mg/ (g.min)) and $\:\beta\:$ is desorption constant (g/mg).

##### Adsorption thermodynamics

In order to identify whether the adsorption mechanism is single-layer physi- or chemisorption, the adsorption thermodynamic parameters, including (Δ*G*^0^), enthalpy (ΔH^0^) and entropy (Δ*S*^0^), must be analyzed. They can be calculated by the following Eqs. 13, 14 and 15 as reported, respectively.

13 $$\:ln\:{\text{K}}_{\text{D}}=\frac{{-\varDelta\:H}^{O}}{RT}+\frac{{\varDelta\:S}^{O}}{R}$$

14 $$\:{\text{K}}_{\text{D}}=\frac{{\text{Q}}_{\text{e}}\:}{{\text{C}}_{\text{e}}\:}$$

15 $$\:{\varDelta\:G}^{O}=-RT\:\text{ln}{\text{K}}_{\text{D}}$$

where *T* is the adsorption temperature (*K*), *K*_D_ is the equilibrium constant, and *R* is the gas constant (8.314 J/mol K)^49,50^.

## Results and discussion

### Attenuated total reflectance infrared spectroscopy (ATR-IR)

**Fig. 1:**
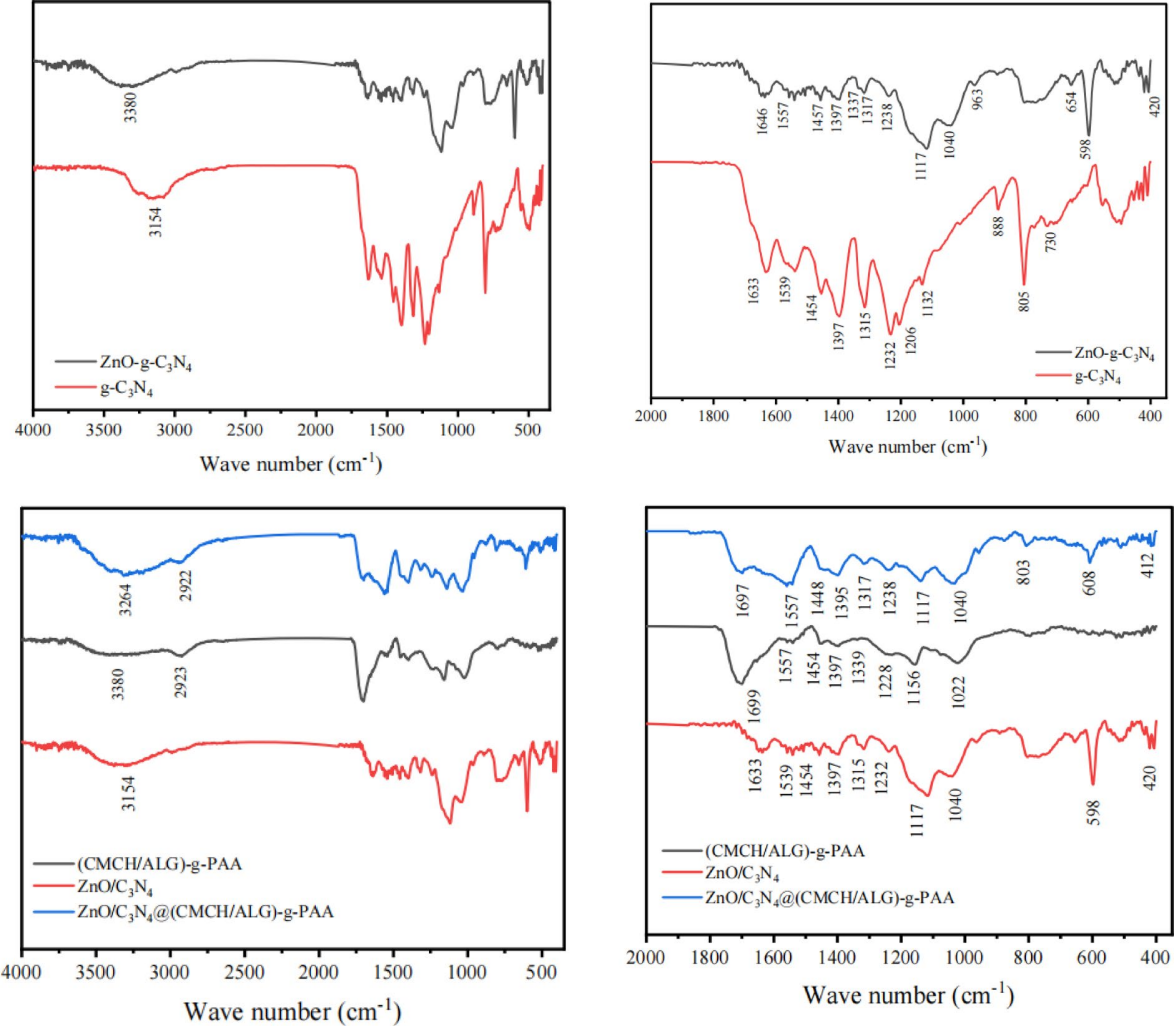
FTIR spectra of g-C_3_N_4_, ZnO-g-C_3_N_4_ nanosheets, (CMCH/ALG)-g-PAA, and ZnO-g-C_3_N_4_ @(CMCH/ALG)-g-PAA copolymers.

The FTIR spectra of g-C_3_N_4_ and ZnO-g-C_3_N_4_ binary hybrid nanosheets were used to further investigate their chemical structures. As seen in (Fig. 1), the bending and stretching modes of N-containing heterocyclic compounds are characterized by a peak at 805 cm^− 1^ and an absorption band in the region between 1000 and 1633 cm^− 1^, respectively, in g-C_3_N_4_^51^. The large peak at 3154 cm^− 1^ region was linked to the hydrogenation of sp^2^-N^52,53^ and the stretching modes of uncondensed amino groups, such as –NH_2_ and = NH^54^.

Ring-sextant out-of-plane bending vibration of the triazine or heptazine units is responsible for the signals at about 805 cm^− 1^^55,56^. The two peaks at 1539 cm^− 1^ and 1633 cm^− 1^ can be ascribed to C = N stretching vibration, while the peaks at 1232 cm^− 1^ and 1454 cm^− 1^ are associated with C–N stretching vibration. There are several absorption maxima for imide and nitride stretching vibrations in the fingerprint area between 1232 and 1633 cm^− 1^^22,57^. The presence of aromatic C-N bonds in g-C_3_N_4_ is indicated by the peaks at 1232 cm⁻¹ and 1315 cm⁻¹.

This Zn–O bond may be the source of the strong FTIR band seen at 420 cm^− 1^ for ZnO-g-C_3_N_4_ binary hybrid nanosheets. When compared to g-C_3_N_4_, the ZnO-g-C_3_N_4_ binary hybrid nanosheets had a shift in the FT-IR peak position, confirming that g-C_3_N_4_ had an extremely strong interaction with ZnO. Furthermore, the ZnO-g-C_3_N_4_ binary hybrid nanosheets exhibit a shift towards 1457 cm^− 1^, 1557 cm^− 1^, and 1646 cm^− 1^ in comparison to g-C_3_N_4_, indicating a weak interaction between C-N and C = N with ZnO. These peaks correspond to C–N and C = N stretching vibrations, respectively. The interaction between g-C_3_N_4_ and ZnO is indicated by the peaks in the 1234 cm^− 1^ − 1633 cm^− 1^ areas being less intense^58–61^. The peak at 805 cm^− 1^ can be attributed to ring-sextant out-of-plane bending vibration of the triazine or heptazine units shifts to 598 cm^− 1^. Thus, the existence of the peaks corresponding to the g-C_3_N_4_ and ZnO in ZnO-g-C_3_N_4_ binary hybrid nanosheets revealed the co-appearance of g-C_3_N_4_ and ZnO in g-C_3_N_4_/ZnO hybrid nanosheets.

CMCH/ALG-g-PAA’s FTIR spectra usually show distinctive peaks that show its structure and changes. Among these peaks is a broad band at 3380 cm^− 1^ that represents the OH and NH groups’ stretching vibrations. C-H stretching vibrations are represented by peaks at about 2923 cm^− 1^. Moreover, the carboxylate groups (-COO) showed two distinctive peaks namely; the symmetrical C = O stretch is a sharp and intense peak at 1699 cm^− 1^ and the asymmetrical C = O stretch appears at 1454 cm^− 1^. The peak at 1557 cm^− 1^ is due to stretching bending of free –NH_2_. The peak at 1390 cm^− 1^ is assigned to (-OH) deformation. The peak at 1339 cm^− 1^ is due to grafting. The peak at 1231 cm^− 1^ is due to C-O-C^62^.

The FTIR of ZnO-g-C_3_N_4_@(CMCH/ALG)-g-PAA copolymer showed similar peaks of (CMCH/ALG)-g-PAA copolymer but with some shits from 3380 cm^− 1^ to 3264 cm^− 1,^ 1699 cm^− 1^ to 1697 cm^− 1^, 1454 cm^− 1^ to 1448 cm^− 1^, 1390 cm^− 1^ to 1317 cm^− 1^, and 1231 cm^− 1^ to 1238 cm^− 1^ due to incorporation of ZnO-g-C_3_N_4_ nanosheets.

The absorption bands in the region from 1000 to 1697 cm^− 1^ and a peak at 803 cm^− 1^ are the characteristic absorptions for the stretching and bending modes of N-containing heterocyclic, respectively are present in ZnO-g-C_3_N_4_@(CMCH/ALG)-g-PAA copolymer. The peak owing to ring-sextant out-of-plane bending vibration of the triazine or heptazine units shifts from 598 cm^− 1^ to 608 cm^− 1^. A new sharp peak reported at 420 cm^− 1^ for ZnO-g-C_3_N_4_ binary hybrid nanosheets could be indexed to the Zn–O bond was shifted to 412 cm^− 1^. These shifts indicated the successful incorporation of ZnO-g-C_3_N_4_ binary hybrid nanosheets into ZnO-g-C_3_N_4_@(CMCH/ALG)-g-PAA copolymer.

### X-ray diffraction patterns

**Fig. 2:**
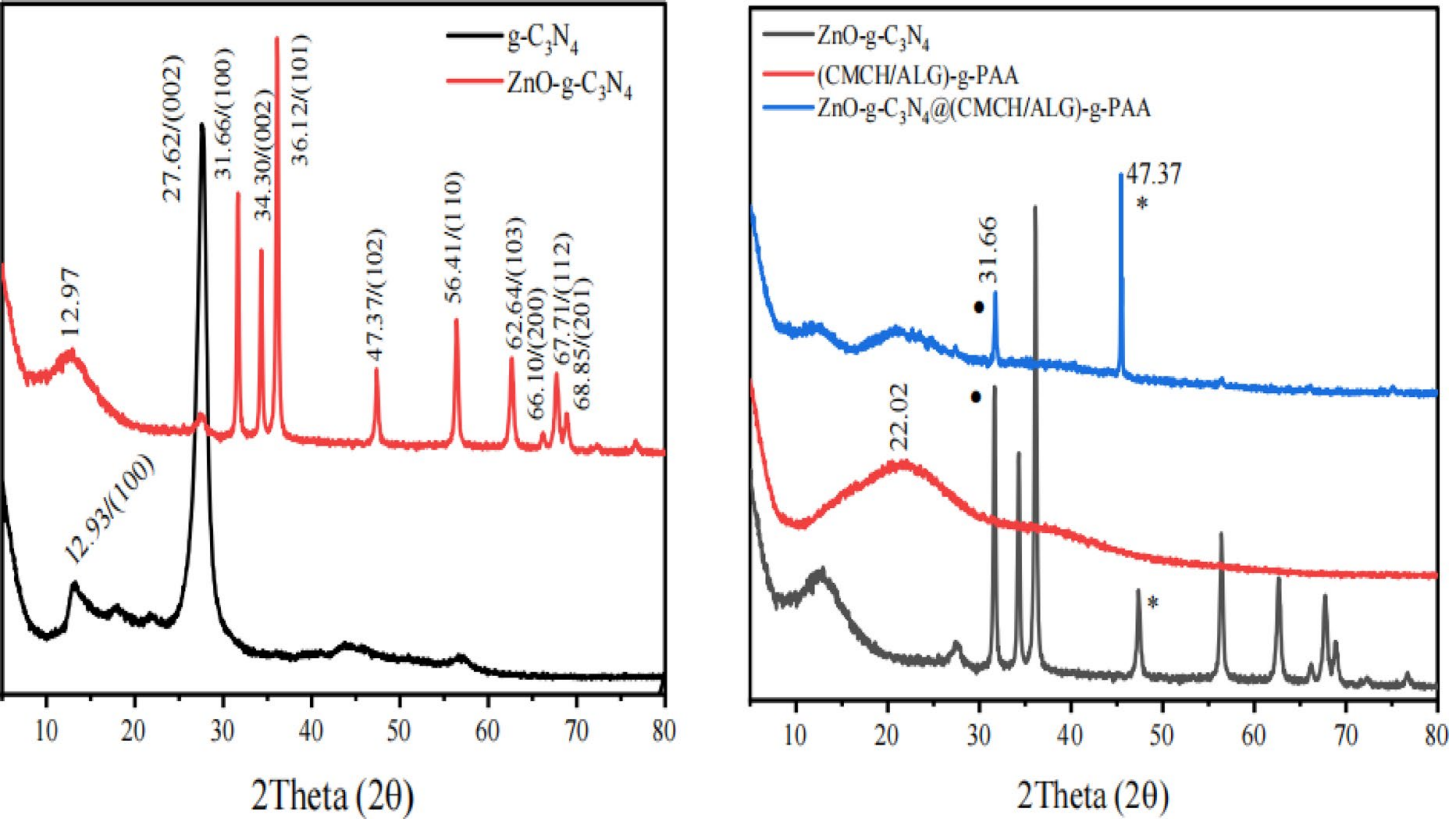
XRD patterns of g-C_3_N_4_, ZnO-g-C_3_N_4_ nanosheets, (CMCH/ALG)-g-PAA, and ZnO-g-C_3_N_4_ @(CMCH/ALG)-g-PAA copolymers.

The g-C_3_N_4_ and ZnO-g-C_3_N_4_ nanosheets’ X-ray diffraction (XRD) patterns are displayed in (Fig. 2). Here, g-C_3_N_4_ and ZnO-g-C_3_N_4_ nanosheets showed two characteristic diffraction peaks. The distinctive interlayer stacking along the c-axis in graphitic carbon nitride is the source of the strong peak at 27.62°, which can be indexed as the diffraction peak of the (002) lattice plane^63,64^. Also, The (100) lattice plane of g-C_3_N_4_ is represented by the weak diffraction peak at 12.94°, which is associated with the heptazine units or the in-plane structural packing motif^65^. The all diffraction peak positions of g-C_3_N_4_ nanosheets are identical when compared to ZnO-g-C3N4. The ZnO-g-C_3_N_4_ nanosheets exhibit the typical diffraction peaks at 2 Theta of 31.66^o^, 34.30°, 36.12°, 47.37°, 56.41°, 62.6° and 67.710°, and 68.85° for ZnO matching to the (100), (002), (101), (102), (110), (103), (112), and (201) crystal planes of ZnO nanoparticles. As shown in (Fig. 2), the (CMCH/ALG)-g-PAA copolymer contains a single peak at 2Theta 22.02^o^ with a semicrystalline nature. Nevertheless, when ZnO-g-C_3_N_4_ nanosheets are integrated into the (CMCH/ALG)-g-PAA copolymer, two additional peaks occur at 2Theta equal 31.60^o^ and 47.37^o^, which correspond to the (100) and (102) diffraction planes of ZnO. Furthermore, (CMCH/ALG)-g-PAA and ZnO-g-C_3_N_4_@(CMCH/ALG)-g-PAA copolymers have estimated crystallinity index percentages (CI%) of 30.78% and 48.15% respectively. The crystallinity index percentage of the (CMCH/ALG)-g-PAA copolymer is increased by 56.39% upon the integration of ZnO-g-C_3_N_4_ nanosheets. Based on these results, ZnO-g-C_3_N_4_ was well in situ integrated into the (CMCH/ALG)-g-PAA copolymer.

### Transmission electron microscope

**Fig. 3:**
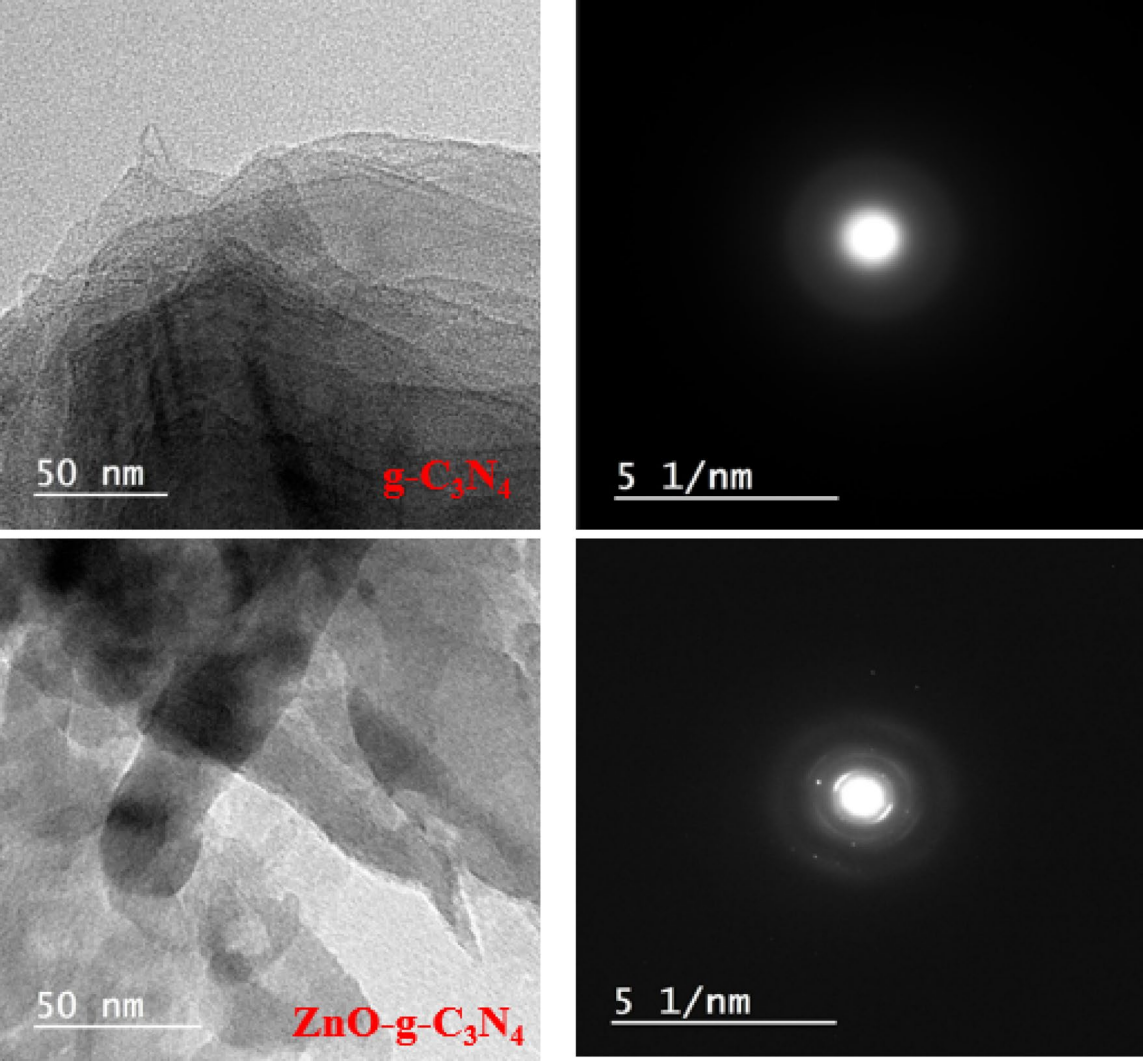
TEM images of g-C_3_N_4_ and ZnO-g-C_3_N_4_ nanosheets and electron diffraction array patterns.

The morphology and structure of g-C_3_N_4_ and ZnO-g-C_3_N_4_ were examined using TEM. TEM pictures of the resultant g-C_3_N_4_ and ZnO-g-C_3_N_4_ are displayed in (Fig. 3). G-C_3_N_4_ nanosheets are porous and have a small, flat, irregular shape in two dimensions^66,67^. TEM images showed thin layer sheets’ stacking orientation. Additionally, TEM images displayed the porous structures’ loose, thin, and soft sheet-like appearance. The porous appearance is due to bubbles from CO_2_, NH_3_, and H_2_O gases that were created during the pyrolysis and condensation events causing this porous architecture. These porous structures are anticipated to be advantageous for increasing the number of active spots on the surface. Adsorption capacity will be improved as a result. Bare ZnO nanoparticles exhibit rod-like structures that are slightly adsorbed on g-C_3_N_4_, as can be seen in the ZnO-g-C_3_N_4_ picture. The amorphous nature of g-C_3_N_4_ and the polycrystalline nature of ZnO-g-C_3_N_4_ are confirmed by the electron diffraction rings array shown in the inset of (Fig. 3)^68,69^.

### Surface morphology

**Fig. 4:**
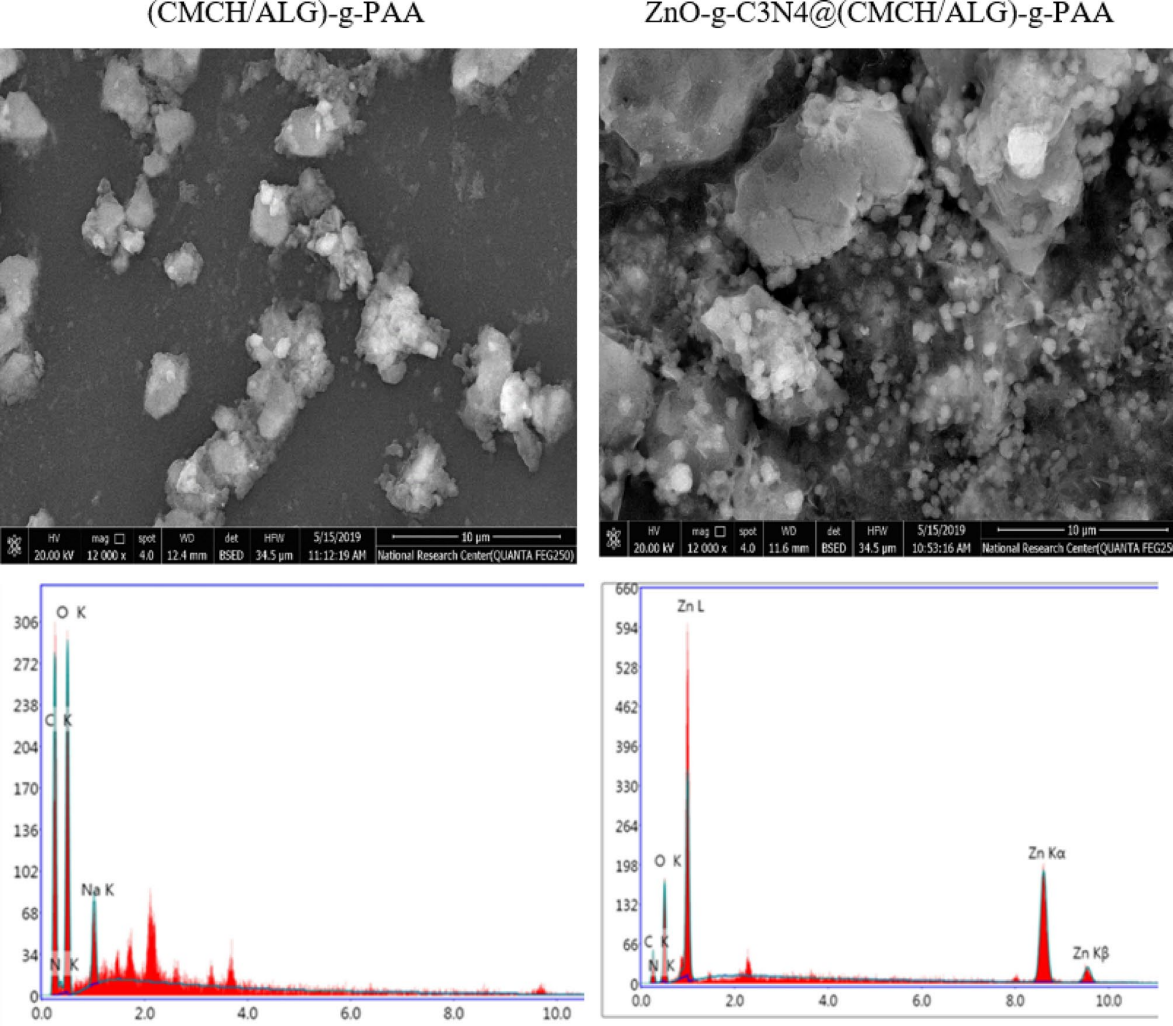
SEM and EDS images of g-C_3_N_4_, ZnO-g-C_3_N_4_, and (CMCH/ALG)-g-PAA, and ZnO-g-C_3_N_4_@(CMCH/ALG)-g-PAA.

SEM was used for additional examination of morphological investigations. The SEM pictures of g-C_3_N_4_, ZnO-g-C_3_N_4_, (CMCH/ALG)-g-PAA, and ZnO-g-C_3_N_4_@(CMCH/ALG)-g-PAA are displayed in (Fig. 4). The lamellar of the g-C3N4-nanosheets is loose and silky lamellar^70^. On the surface of graphitic carbon nitride, ZnO-g-C_3_N_4_ exhibits a typical stacked multilayered sheet-like morphology that corresponds to the in-plane structural packing pattern of either triazine rings or heptazine rings building blocks. The ZnO-g-C_3_N_4_ sample shows clearly defined ZnO nanoparticles. This outcome confirms that the ZnO-g-C_3_N_4_ sample’s sharp X-ray diffraction pattern was caused by its high crystalline quality. When ZnO nanoparticles are present, a stalked, interconnected network forms. The (CMCH/ALG)-g-PAA appears as tiny deposits of uneven patches on the CMCH/ALG. However, ZnO-g-C_3_N_4_@(CMCH/ALG)-g-PAA showed the embedded lamellar ZnO-g-C_3_N_4_ nanosheets as presented in (Fig. 4).

Correspondingly, the EDS spectra patterns of g-C_3_N_4_, ZnO-g-C_3_N_4_, (CMCH/ALG)-g-PAA, and ZnO-g-C_3_N_4_@(CMCH/ALG)-g-PAA are depicted in (Fig. 4) and atomic ratios. of SEM-EDS elemental mapping are included in (Table 1). G-C_3_N_4_ nanosheets showed three elements including C, O, and N. On the other hand, ZnO-g-C_3_N_4_ showed that it was composed of the elements O, C, N, and Zn. It was revealed that the elements O, N, C, and Zn were clearly defined and contrasted sharply. The ZnO elements are effectively and uniformly distributed throughout. The outcomes validated the ZnO-g-C_3_N_4_ nanocomposite’s successful construction^71^. (CMCH/ALG)-g-PAA exposed three elements C, N, and O and The ZnO-g-C_3_N_4_@(CMCH/ALG)-g-PAA copolymer’s EDS spectra revealed a contrast between the elements C, N, O, and Zn. ZnO-g-C_3_N_4_ was confirmed to be well-embedded into the (CMCH/ALG)-g-PAA copolymer by the EDS data.

**Table 1:** SEM-EDS elemental mapping and its atomic ratios.

Sample types	Atomic ratios			
	C	O	*N*	Zn
g-C_3_N_4_	41.77	26.46	26.77	0
ZnO@g-C_3_N_4_	35.24	25.19	24.32	15.25
(CMCH/ALG)-g-PAA	53.77	40.77	4.54	0
ZnO-g-C_3_N_4_@(CMCH/ALG)-g-PAA	47.84	42.32	5.73	4.1

### Thermal analysis

**Fig. 5:**
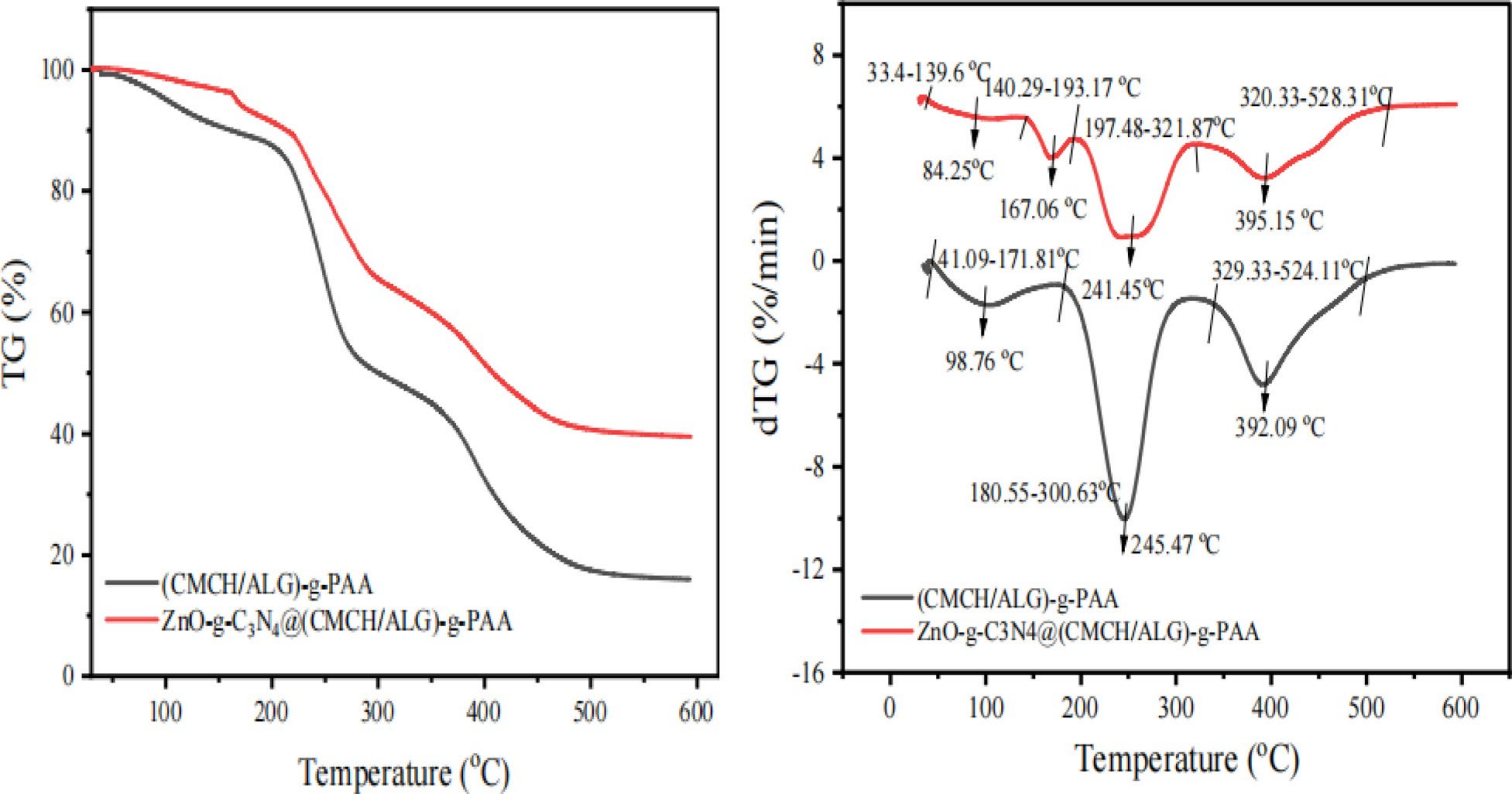
TG% and dTG% of (CMCH/ALG)-g-PAA and ZnO-g-C_3_N_4_@(CMCH/ALG)-g-PAA.

Thermo gravimetric curves of (CMCH/ALG)-g-PAA and ZnO-g-C_3_N_4_@(CMCH/ALG)-g-PAA are displayed in (Fig. 5). There was a noticeable weight loss that started as soon as the heating started. The three the decomposition phases of (CMCH/ALG)-g-PAA are 41.09-171.81 ˚C, 180.55–30,063 ˚C, and 329.33-524.11 ˚C with weight loss % (10.57, 50.05, and 83.25%), respectively as shown in (Table 2). The initial decomposition is due to desorption of moisture as hydrogen-bound water to (CMCH/ALG)-g-PAA. The second and third decomposition phases are due to depolymerization of graft chains to (CMCH/ALG) substrate which occurred at starting temperatures 180.55 ˚C and 329.33 ˚C with evolving of water vapors, carbon dioxide, and organic volatiles gases such as methane^72^.

On other hand, ZnO-g-C_3_N_4_@(CMCH/ALG)-g-PAA copolymer exhibits four degradation phases 34.95 ˚C -194.04 ˚C, 194.04 ˚C -239.26 ˚C, 239.26 ˚C -311.21 ˚C, 320.33 ˚C -528.31 ˚C with weight loss percentages of 8.05%, 17.03%, 35.73%, and 59.82%: The initial degradation temperature of (CMCH/ALG)-g-PAA copolymer (171.81 ˚C) has shifted into 194.04 ˚C up on incorporation of ZnO-g-C_3_N_4_ indicating thermal stability of ZnO-g-C_3_N_4_@(CMCH/ALG)-g-PAA. Furthermore, the occurrence of four thermal stages with low weight loss percentages than those of (CMCH/ALG)-g-PAA, this means that ZnO-g-C_3_N_4_@(CMCH/ALG)-g-PAA is more thermal stable than (CMCH/ALG)-g-PAA. Also, Table 2 shows thermal kinetic parameters of both (CMCH/ALG)-g-PAA and ZnO-g-C_3_N_4_@(CMCH/ALG)-g-PAA copolymers using Coats-Redfern equation to obtain total activation energy, *n* was taken from 0.0 to 3.0 with 0.5 increments. For each value of *n*, (*R*^2^) was evaluated. The activation energies were estimated with the best value of *R*^2^. The total activation energy of ZnO-g-C_3_N_4_@(CMCH/ALG)-g-PAA copolymer (Ʃ -0.1158 J mol^− 1^) is two-fold higher than (CMCH/ALG)-g-PAA copolymer (Ʃ -0.05939 J mol^− 1^).

**Table 2:** Thermal analysis and kinetics parameters of TG curves of (CMCH/ALG)-g-PAA and ZnO-g-C_3_N_4_@(CMCH/ALG)-g-PAA copolymers.

(CMCH/ALG)-g-PAA								
Step	Temp. range ^o^C	Max. weight loss ^o^C	*R* ^2^		*RSS*		*n*	*E*a (J mol^− 1^)
^1st^	41.09-171.81	98.76	0.9983		1.16 × 10^− 12^		0.5	-0.0107
^2nd^	180.55-300.63	245.47	0.9918		6.11 × 10^− 13^		0.5	-0.0112
^3rd^	329.33-524.11	392.09	0.9783		2.37 × 10^− 11^		0	-0.0374
								Ʃ -0.05939

ZnO-g-C_3_N_4_@(CMCH/ALG)-g-PAA								
Step	Temp. range ^o^C	Max. weight loss ^o^C	*R* ^2^		*RSS*		*n*	*E*a (J mol^− 1^)
^1st^	34.95-194.04	-	0.9995		2.01 × 10^− 12^		0	-0.0226
^2nd^	194.04-239.26	222.01	0.9936		2.21 × 10^− 14^		0.5	-0.0097
^3rd^	239.26-311.21	281.12	0.9935		6.87 × 10^− 14^		0.5	-0.0104
^4^th	320.33-528.31	364.43	0.9916		4.25 × 10^− 13^		0.5	-0.0731
								Ʃ -0.1158

### Swelling ratios studies

#### Effect of pH and saline on swelling and its kinetics

The hydrogel was swelling essential for dye removal. This swelling makes it easier for dissolved dyes to bind to the accessible polymer chains by granting access to adsorption sites within the polymer network, which are only made available once the hydrogel opens up in the water. The adsorption of dye molecules from aqueous solutions through a variety of interactions, including hydrogen bonding, van der Waals forces, and electrostatic attraction, is made possible by the expansion of the 3D network, which reveals the hydrophilic functional groups and pore structure of the hydrogel.

**Fig. 6:**
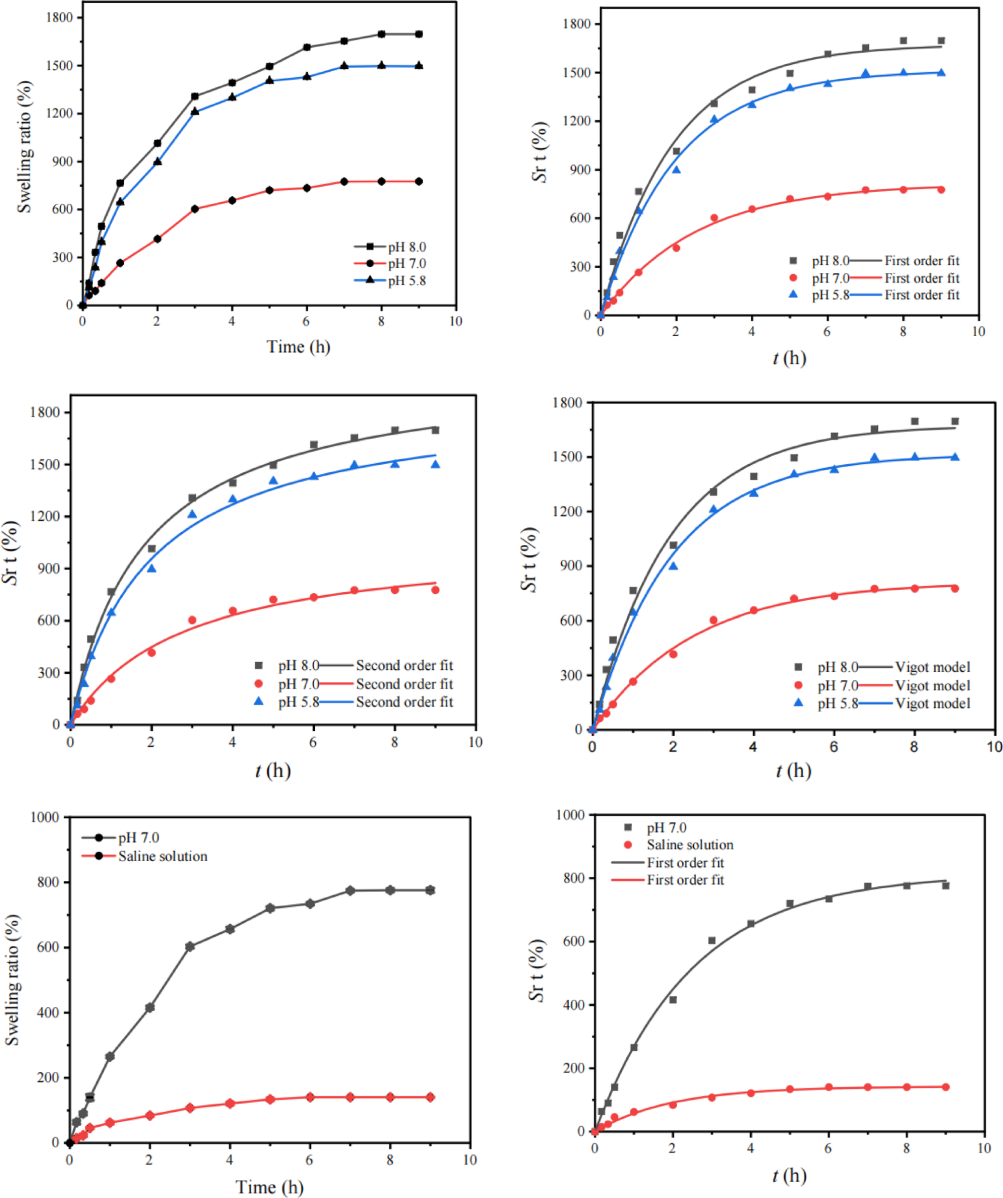

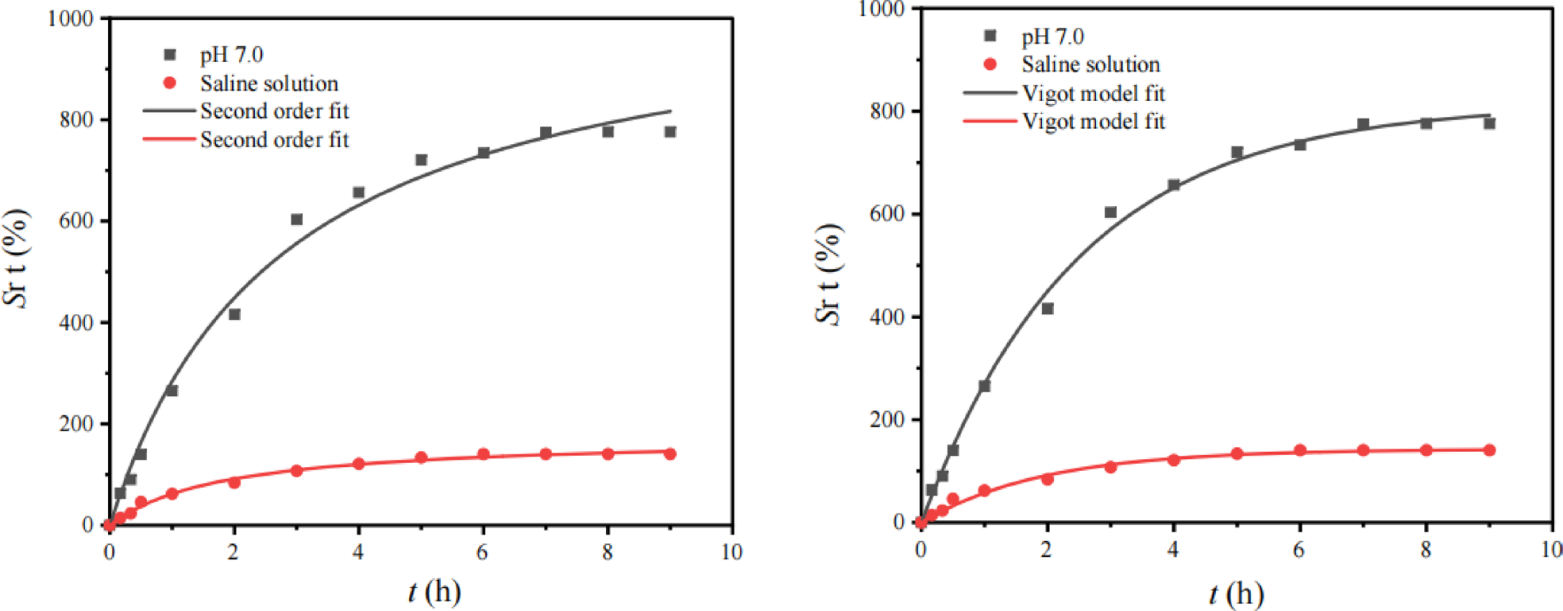
Swelling ratios % and its kinetic of (CMCH/ALG)-g-PAA copolymer at different pH media using first order, second order, and Vigot models.

 Figure 6 displays the pH-sensitive swelling behavior of (CMCH/ALG)-g-PAA copolymer at different range of pH. The swelling equilibrium appeared on an average of 9 h. The swelling behavior of synthesized (CMCH/ALG)-g-PAA copolymer was found to be pH dependent, which was governed by the functional moieties present in the polymeric chain such as free amino, carboxyl groups, and hydroxyl groups. As shown in (Fig. 6), high swelling was observed at two pH levels 5.8 and at 8.0 which is explained by the following mechanisms: in low pH 5.8, the percentage swelling was governed by the -NH_2_ moieties present in the (CMCH/ALG)-g-PAA copolymer. In excess of H^+^ ions, this functional group got protonated resulting in mutual repulsion between two polymeric chains, thus allowing more spaces for water. In alkaline condition, the percentage of water uptake was solely controlled by the carboxylic acid groups present in hydrogel chain. In excess of OH–ions, COOH existed as carboxylate ions (COO^−^). These negative charge ions induced repulsion among adjacent carboxylate ions of polymeric chains and anions counter part of alkaline buffer solution. Hence, huge intermolecular spaces were available for water^73,74^. The maximum swelling ratios were 1697.10 ± 5.0%, 1496.24 ± 5.5%, and 775.99 ± 6.2% match to pH 8.0, pH 5.8, and pH 7.0.

The swelling kinetics of (CMCH/ALG)-g-PAA copolymer in buffer solution at various pH as well as in saline solution are represented by Pseudo-first, second-order reactions, and Vigot model (Fig. 6). The swelling ratios readings quite well fit with the aforementioned models. The results of kinetic parameters at various pH are found in (Table 3). The swelling of (CMCH)-g-PAA follows the pseudo-first-order reaction with higher correlation coefficients (*R*^*2*^f ≥ 0.99) and smaller nonlinear error functions, the Chi-square (χ^2^) rather than second order despite higher correlation coefficient. Additionally, Table 3 displays that the pseudo-first-order reactions predicted equilibrium swelling ratios (*Sr*e, f, fit %) are reasonably close to the actual ratios. One of the most crucial technical aspects of (CMCH/ALG)-g-PAA composite is their swelling rate. Free-absorbency capacity measurements are typically performed at successive time intervals to acquire the profile of swelling capacity versus time of (CMCH/ALG)-g-PAA composite sample, which may be used to calculate the swelling rate. The swelling ratios values derived from the data are fitted into a Voigt model and showed good fitting with model.

The swelling capacity at equilibrium represents the greatest water-holding capacity (*S*_e, V_) is reasonably close to actual data as shown in (Table 3). Another Vigot`s crucial parameter is rate parameter (*r*,V) is the time required to reach 0.63 of the equilibrium swelling. It is noticed that pH has effect on (*r*,V). The decreasing order of (*r*,V) is 2.48 ± 0.120 h, 1.96 ± 0.08 h, and 1.90 ± 0.14 h which match to pH 7.0, pH 5.8, and pH 8.0 as shown in (Table 3). When compared to the values obtained in buffer solution at pH 7.0, the superabsorbent’s swelling capacity in saline solution was noticeably reduced from 775.9 ± 6.24% to 140.5 ± 1.24% as shown in (Fig. 6). The non-effective anion–anion electrostatic repulsion caused by the screening effect of the extra cations is the cause of the swelling observed in the ionic (CMCH/ALG)-g-PAA copolymer.

**Table 3:** Swelling ratios kinetics parameters of (CMCH/ALG)-g-PAA copolymer at diverse pH using first order, second order, and vigot models.

Parameters	pH 5.8	pH 7.0	pH 8.0
*k*_*1*,*f*_	0.525 ± 0.03	0.402 ± 0.01	0.508 ± 0.02
*Sr*_e_, exp %	1496.2 ± 5.50	775.9 ± 6.24	1697.1 ± 5.0
*Sr*_e, f_, fit %	1474.4 ± 35.4	813.8 ± 13.0	1612.31 ± 24.67
*R* ^2^_f_	0.9912	0.9971	0.9970
χ^2^_f_	0.378	0.298	0.107
*k*_2_, _s_	2.70 × 10^− 4^±2.09	3.40 × 10^− 4^±5.32	2.71 × 10^− 4^±2.84
*Sr*_e, S_, fit %	2057.6 ± 37.3	1066.2 ± 44.7	1889.7 ± 47.5
*R* ^2^_s_	0.9973	0.9925	0.9955
χ^2^_s_	0.114	0.783	0.162
*Sr*_e_, _V_	1515.8 ± 19.4	813.8 ± 13.0	1674.5 ± 35.6
r, _V_	1.96 ± 0.08	2.48 ± 0.120	1.90 ± 0.14
χ^2^_V_	0.378	0.298	0.107
*R* ^2^_V_	0.9912	0.9971	0.9970

Additionally, because of the difference in the concentration of mobile ions between the copolymer and aqueous phases, there is a reduced osmotic pressure difference between the (CMCH/ALG)-g-PAA copolymer network and the external solution. The swelling ratios % data are well fitted with first order and are not fitted with second order. Because of the simulated *Sr*_e, S_, fit % (1889.7 ± 47.5) is further far from experimental one, *Sr*e, exp % =140.50 ± 1.24 as shown in T(able 4). On the other hand, the swelling ratios % data are well fitted with Vigot model and higher R^2,^_V =_0.9902 and the swelling capacity at equilibrium (*S*r_e_, _V_), is fairly similar to the actual values and the rate parameter (r, V) is 1.93 ± 0.15 h as shown in (Table 4).

**Table 4:** Swelling ratios kinetics parameters of (CMCH/ALG)-g-PAA copolymer in saline solution.

Parameters	Saline solution
*k*_*1*_, _f_	0.517 ± 0.04
*Sr*_e_, exp %	140.50 ± 1.24
*Sr*_e, f_, fit %	142.6 ± 3.22
*R* ^2^_f_	0.9902
χ^2^_f_	0.305
*k*_*2*_, _s_	3.09 × 10^− 3^±2.95 × 10^− 5^
*Sr*_e, S_, fit %	1889.7 ± 47.5
*R* ^2^_s_	0.9926
χ^2^_s_	0.925
*Sr*_e_, _V_	142.6 ± 3.24
r, _V_	1.93 ± 0.15
χ^2^_V_	0.305
*R* ^2^_V_	0.9902

### Evaluation of adsorption capacity of ZnO-g-CN_4_(3%)@(CMCH/ALG)-g-PAA

#### Effect of ZnO-g-CN_4_ contents

**Fig. 7:**
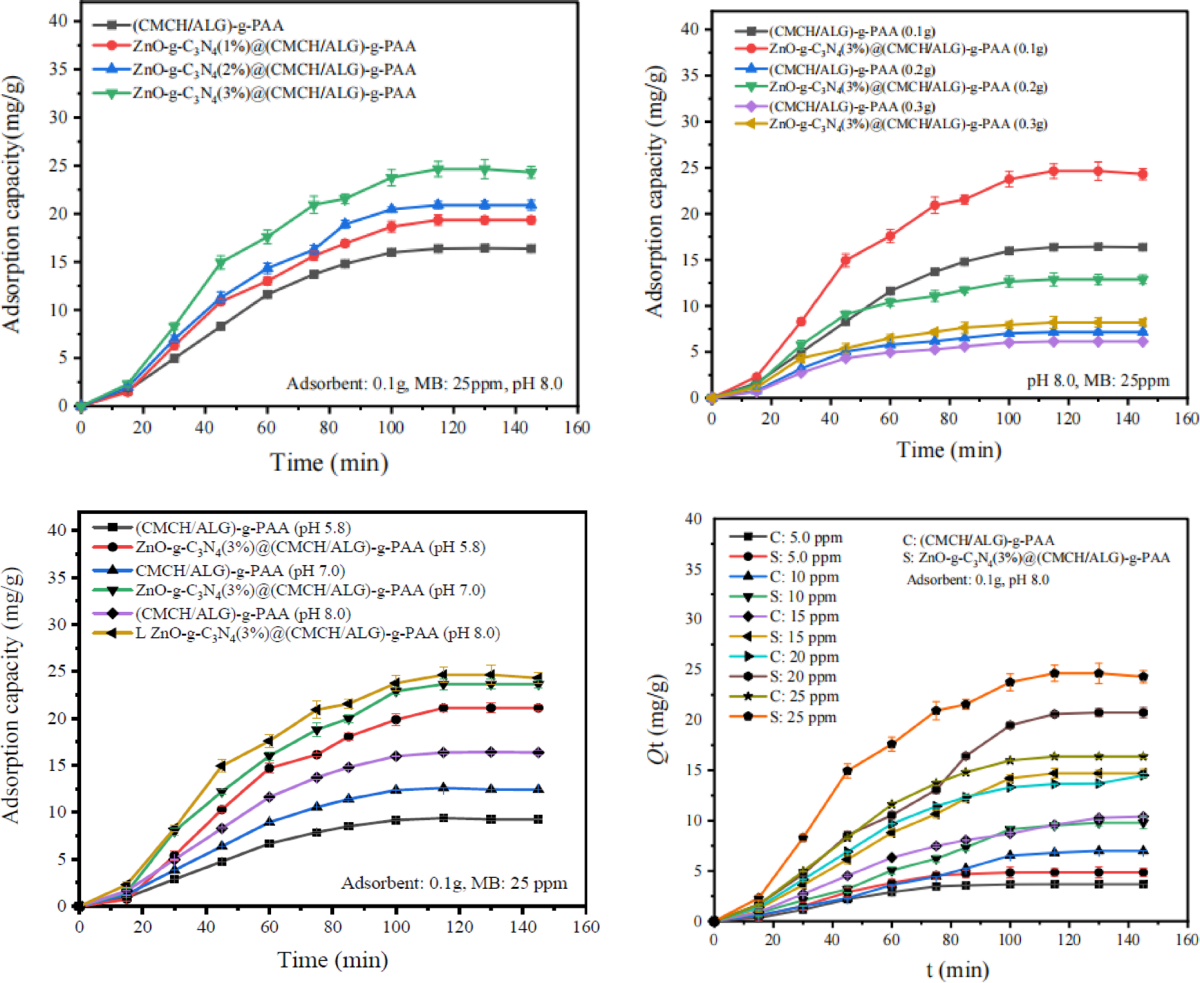
Evaluation of adsorption capacity of ZnO-g-C_3_N_4_(3%)@(CMCH/ALG)-g-PAA.

The effect of embedded ZnO-g-C_3_N_4_ contents in (CMCH/ALG)-g-PAA on the adsorption of MB dye was studied over a weight gradient (1%, 2%, and 3% based on weight of CMCH/ALG)-g-PAA) adsorbent and the results are presented in (Fig. 7). An increase in the amount of dosage from 1% to 3% increases the adsorption capacity from 19.34 ± 0.39 mg/g to 24.30 ± 0.61 mg/g. ZnO-g-C_3_N_4_(3%)@(CMCH/ALG)-g-PAA’s adsorption mechanism of MB was primarily based on three interactions: π-π stacking orientations, electrostatic interactions, and intermolecular hydrogen bonds. It is common for the aryl structures to create π-π stacking interactions in edge-to-face and/or face-to-face locations. When 3% ZnO-g-C_3_N_4_ is embedded in CMCH/ALG)-g-PAA polymer, π-π stacking with MB is formed, which makes it possible for MB to be adsorbed onto the polymer surface. Furthermore, the surface of ZnO-g-C3N4@(CMCH/ALG)-g-PAA nanocomposite has a significant number of carboxyl, amino, and hydroxyl groups at 3% ZnO-g-C_3_N_4_. This includes an O atom, which acts as an excellent hydrogen acceptor and contributes to form hydrogen bonds with N and S ions in MB molecules^21^.

#### Effect of ZnO-g-CN_4_(3%)@(CMCH/ALG)-g-PAA adsorbent dose

The dosage of the ZnO-g-C_3_N_4_(3%)@(CMCH/ALG)-g-PAA adsorbent is a crucial factor that needs to be assessed and adjusted. The adsorption capacity for MB dye increased when the ZnO-g-C_3_N_4_(3%)@(CMCH/ALG)-g-PAA adsorbent dosage increased from 0.1 to 0.3 g/L, according to the results (Fig. 7). This could be because the active sites on the surface of each adsorbent became unsaturated. However, as the adsorbent dosage was increased, the rate of removal of MB dye increased as well. This is as expected since, with a higher amount of the adsorbent, the overall surface area is increased and therefore there are more active sites accessible for the adsorption^75^.

Hamid Safarzadeh et al., 2023 had created methacrylic acid (MAA) and acrylamide (AAm) onto the sodium alginate (SA) to remove malachite green (MG) from aqueous solution. The maximum adsorption capacity was 95.91 mg/g at a pH of 7, adsorbent polymer concentration of 1.5 g/L, a contact time of 90 min, and MG concentration of 10 mg/L^76^. Monu VermaIn 2022, et al. synthesized a graphene oxide-chitosan nanocomposite functionalized with ethylenediaminetetraacetic acid (EDTA). The adsorption of MB was based on the Langmuir isotherm model, with a maximum adsorption capacity of 141 ± 6.60 and mg g^− 1^ 15 at solution pH of 5.10 and 8.30. The initial concentrations of MB dye were 100 mg L^− 1^ at 240 min of contact time and an adsorbent dose of 50 mg (in 40 mL solution)^15^.

#### Effect of solution pH

One significant factor influencing the entire adsorption process is the pH of the solution. It has the ability to change the adsorbent’s surface charge. As a result, there will be a range of interactions between the organic dye and the adsorbent composite, from attraction to repulsion. Thus, the adsorption capacity will decline or increase with increasing the pH, depending on whether the organic dye is cationic or anionic and its interaction with ZnO-g-C_3_N_4_(3%)@(CMCH/ALG)-g-PAA adsorbent^77^. The MB dye’s structure is distinguished by the existence of cationic functional groups. ZnO-g-C_3_N_4_(3%)@(CMCH/ALG)-g-PAA adsorbent’s surface charge becomes positively charged at acidic pH (pH = 5.8), which reduces the attraction between the cationic dye and the ZnO-g-C_3_N_4_(3%)@(CMCH/ALG)-g-PAA adsorbent and, as a result, lowers the adsorption capacity. However, at alkaline pH (pH 8.0), the ZnO-g-C_3_N_4_(3%)@(CMCH/ALG)-g-PAA adsorbent becomes negatively charged, therefore the cationic MB dye could be electrostatically attracted leading into high adsorption capacity. The increasing order is 21.11 ± 0.31 mg/g, 23.64 ± 0.43 mg/g, and 24.30 ± 0.61 mg/g that match to pH 5.8, pH 7.0, and pH 8.0, respectively (Fig. 7). Ch. Jamkhokai Mate and Sumit Mishra (2020) have reported a maximum adsorption 5.6 mg/g of Remazol Brilliant Blue R (RBBR) dye at 80 mg/L dye concentration and pH 6 for a cross-linked Jhingan gum hydrogel was synthesized using borax as a cross-linker through microwave assisted technique^73^.

#### Effect of initial MB concentrations

 Figure 7 illustrates the adsorption capacity as a function of the initial MB concentration, which ranges from 5 to 25 mg/L. An increase in the initial MB concentration was observed to boost the adsorption capacity. The adsorption capacity was 24.30 ± 0.61 mg/g at the starting concentration of 25 mg/L as displayed in (Fig. 7). Accordingly, there is a greater concentration gradient and a stronger driving force for adsorption of the MB dye at higher starting concentrations and fixed amount of ZnO-g-C_3_N_4_(3%)@(CMCH/ALG)-g-PAA adsorbent (0.3 g/L) and adjusted pH of 8.0. This provides the MB dye greater opportunities to get into contact with the ZnO-g-C_3_N_4_ (3%)@(CMCH/ALG)-g-PAA adsorbent leading into a higher adsorption capacity will result from the attachment of more MB dye to the active sites on the adsorbent surface. According to this, ZnO-g-C_3_N_4_ (3%)@(CMCH/ALG)-g-PAA copolymer is a great adsorbent that can sustain a steady removal rate by boosting its adsorption capacity as the starting concentration increases. The gum Acacia capped polyaniline-based nanocomposite hydrogel was fabricated by T. Jayaramudu et al. (2021) had a maximum adsorption capacity of MB 35.41 mg/g at pH 10 with a dye concentration of 10 mg L^− 1^., 300 min^78^.

#### Adsorption kinetics and modeling

**Fig. 8:**
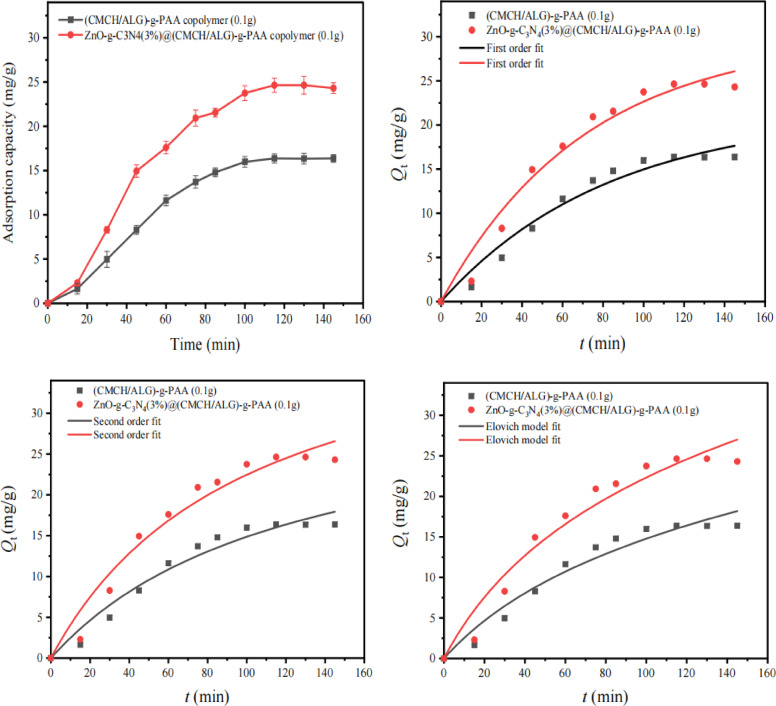
Adsorption rate curves of MB dye: experimental conditions: pH 8.0, dye conc. 25 mg/L, (CMCH/ALG)-g-PAA and ZnO-g-C_3_N_4_(3%)@(CMCH/ALG)-g-PAA adsorbents doses (0.3 g/L).

Investigation of adsorption Kinetics is significant because it allows determining the mechanism of adsorption as well as the rate of adsorption, which is one of the requirements for an adsorbent’s efficiency. The change in the amount of MB dye adsorbed (*Q*t) by ZnO-g-C_3_N_4_(3%)@(CMCH/ALG)-g-PAA in comparison with (CMCH/ALG)-g-PAA adsorbents as a function of time is displayed in (Fig. 8). In the early stages of adsorption, MB dye exhibits a high rate of adsorption. The majority of adsorption occurs in less than 50 min, indicating a rapid rate of dye adsorption by ZnO-g-C_3_N_4_(3%)@(CMCH/ALG)-g-PAA and (CMCH/ALG)-g-PAA adsorbents, respectively. In order to analyze the adsorption kinetics of MB dye by ZnO-g-C_3_N_4_(3%)@(CMCH/ALG)-g-PAA and (CMCH/ALG)-g-PAA adsorbents, the pseudo-first order, pseudo-second order, and Elovich equation are tested. The result of fitting is listed in (Table 5). Despite all the experimental data had higher correlation coefficient values (*R*^2^ > 0.9), but the data have better compliance with the first order kinetic model compared to the pseudo second-order model. The *Q*_e, f_, fit % values 21.3 ± 2.49 and 29.9 ± 2.74 from the first-order model for (CMCH/ALG)-g-PAA and ZnO-g-C_3_N_4_(3%)@(CMCH/ALG)-g-PAA adsorbents is more in line with the experimental *Q*_e_, exp % values 16.3 ± 0.43 and 24.3 ± 0.611 for (CMCH/ALG)-g-PAA and ZnO-g-C_3_N_4_(3%)@(CMCH/ALG)-g-PAA adsorbents, respectively.

In general, chemisorption is applied using the Elovich equation. The equation has been shown to apply to a broad variety of slow adsorption rates and has been applied well to some chemisorption processes. The same equation is frequently applicable to systems with heterogeneous adsorbing surfaces. The experimental data is consistent with Elovich model fitting with high *R*^2^ and the initial adsorption rate constant ($\:\alpha\:)$ of ZnO-g-C_3_N_4_(3%)@(CMCH/ALG)-g-PAA (0.482 ± 0.107 mg g^− 1^ min^− 1^) is higher than that of (CMCH/ALG)-g-PAA adsorbent (0.282 ± 0.05 mg g^− 1^ min^− 1^) and the desorption constant ($\:\beta\:$) of (CMCH/ALG)-g-PAA adsorbent (0.079 ± 0.02 g min^− 1^) is higher than that of ZnO-g-C_3_N_4_(3%)@(CMCH/ALG)-g-PAA (0.062 ± 0.016 *g* min^− 1^) indicating that ZnO-g-C_3_N_4_(3%)@(CMCH/ALG)-g-PAA is highly efficient adsorbent for removal of MB dye.

**Table 5:** Adsorption kinetics modeling parameters at pH 8.0, MB concentration 25 mg/L, and adsorbent dose 0.1 g using first order, second order, and Elovich models.

Parameters	(CMCH/ALG)-g-PAA	ZnO-g-C_3_*N*_4_(3%)@(CMCH/ALG)-g-PAA
*k*_1_, _f_	0.012 ± 0.02	0.014 ± 0.00
*Q*_e_, exp (mg g^− 1^)	16.3 ± 0.43	24.3 ± 0.611
*Q*_e, f_, fit (mg g^− 1^)	21.3 ± 2.49	29.9 ± 2.74
*R*^2^_f_ (min^− 1^)	0.9689	0.9712
χ^2^_f_	0.133	0.267
*k*_2_, _s_ (g mg^− 1^ min^− 1^)	2.44 × 10^− 4^±1.29 × 10^− 4^	2.23 × 10^− 4^±1.02 × 10^− 4^
*Q*_e, S_, fit (mg g^− 1^)	33.1 ± 6.29	44.87 ± 7.05
R^2^_s_	0.9629	0.9642
χ^2^_s_	0.158	0.332
*α* (mg g^− 1^ min^− 1^)	0.282 ± 0.05	0.482 ± 0.107
*β* (g min^− 1^)	0.079 ± 0.02	0.062 ± 0.016
χ^2^, _V_	0.182	0.398
*R*^2^, _V_	0.9573	0.9571

#### Adsorption isotherm

**Fig. 9:**
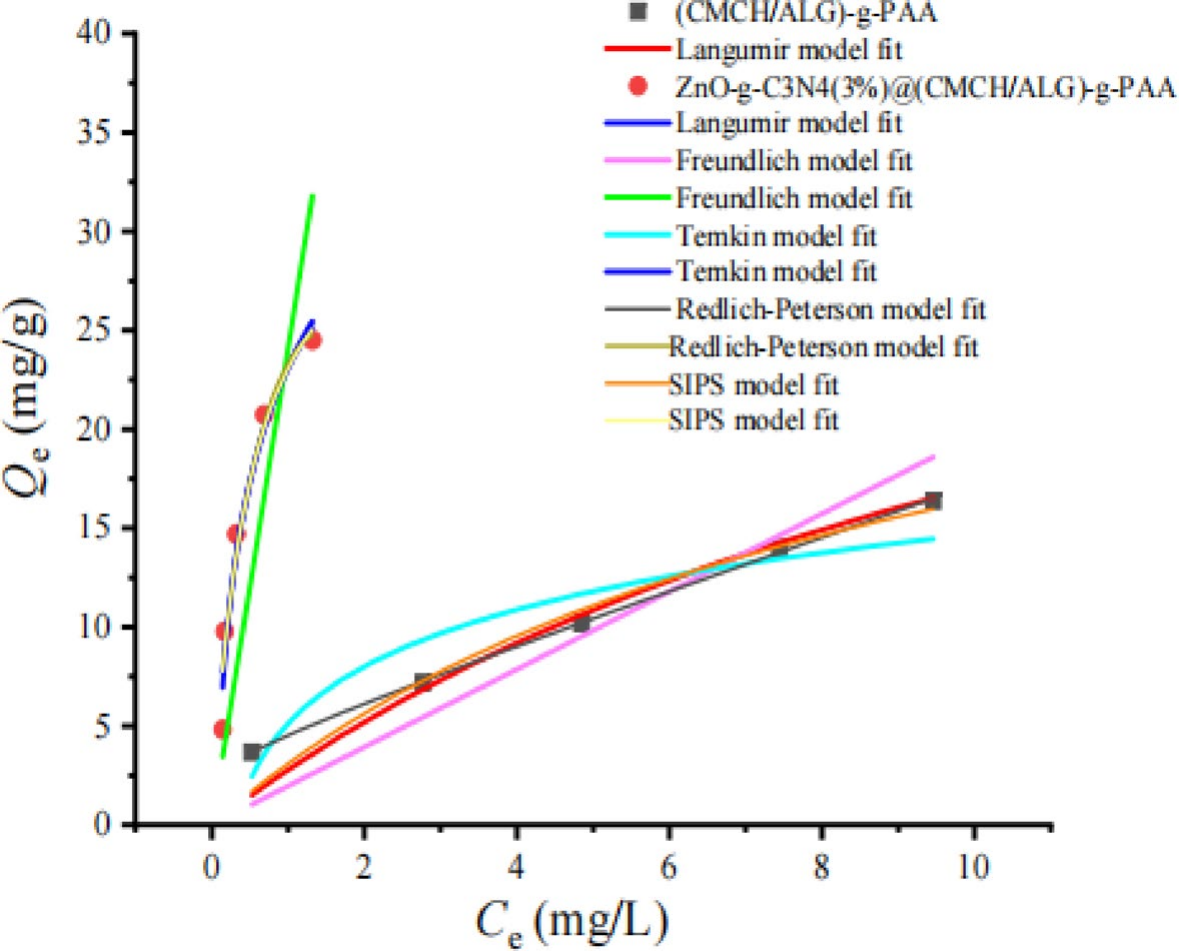
Adsorption isotherm modeling of MB dye by of ZnO-g-C_3_N_4_(3%)@(CMCH/ALG)-g-PAA in comparison with (CMCH/ALG)-g-PAA adsorbents.

In order to further confirm the adsorption mechanism, the most commonly used model equations, Langmuir (Eq. 16), Freundlich (Eq. 17), Temkin (Eq. 18), Redlich Peterson (Eq. 19)^79^, Sips (Eq. 22)^80^, and Fritz–Schlunder (F–S) isotherm (Eq. 23)^81^, respectively were applied to explain the adsorption process. A comparison of nonlinear fitted curves of MB dye adsorbed by ZnO-g-C_3_N_4_(3%)@(CMCH/ALG)-g-PAA and (CMCH/ALG)-g-PAA adsorbents from experimental data is shown in (Fig. 9). The coefficients of determination (*R*^2^) and isotherm parameters from nonlinear regressive method were listed in (Table 6).

According to the Langmuir model, monolayer sorption takes place on a solid surface with homogeneous, identical sites. Additionally, it implies that after the active sites are saturated with dye molecules, no more adsorption occurs.

16 $$\:{\text{Q}}_{\text{e}}=\frac{{\text{(Q}}_{\text{max}}\:{\text{K}}_{\text{L}}{\text{C}}_{\text{e}}\text{)}}{1+{\text{C}}_{\text{e}}\:\:{\text{K}}_{\text{L}}}$$

Where *Q*_e_ is the quantity of the adsorbed adsorbate per unit mass of the adsorbent (mg/L), *C*_e_ is the adsorbate equilibrium concentration (mg/L). *K*_*L*_ = a constant which is related to the affinity existing between the adsorbate and the adsorbent. *Q*_max_ (mg g^− 1^) is the saturation ability of the monolayer which is expressed theoretically.

**Scheme 3:**
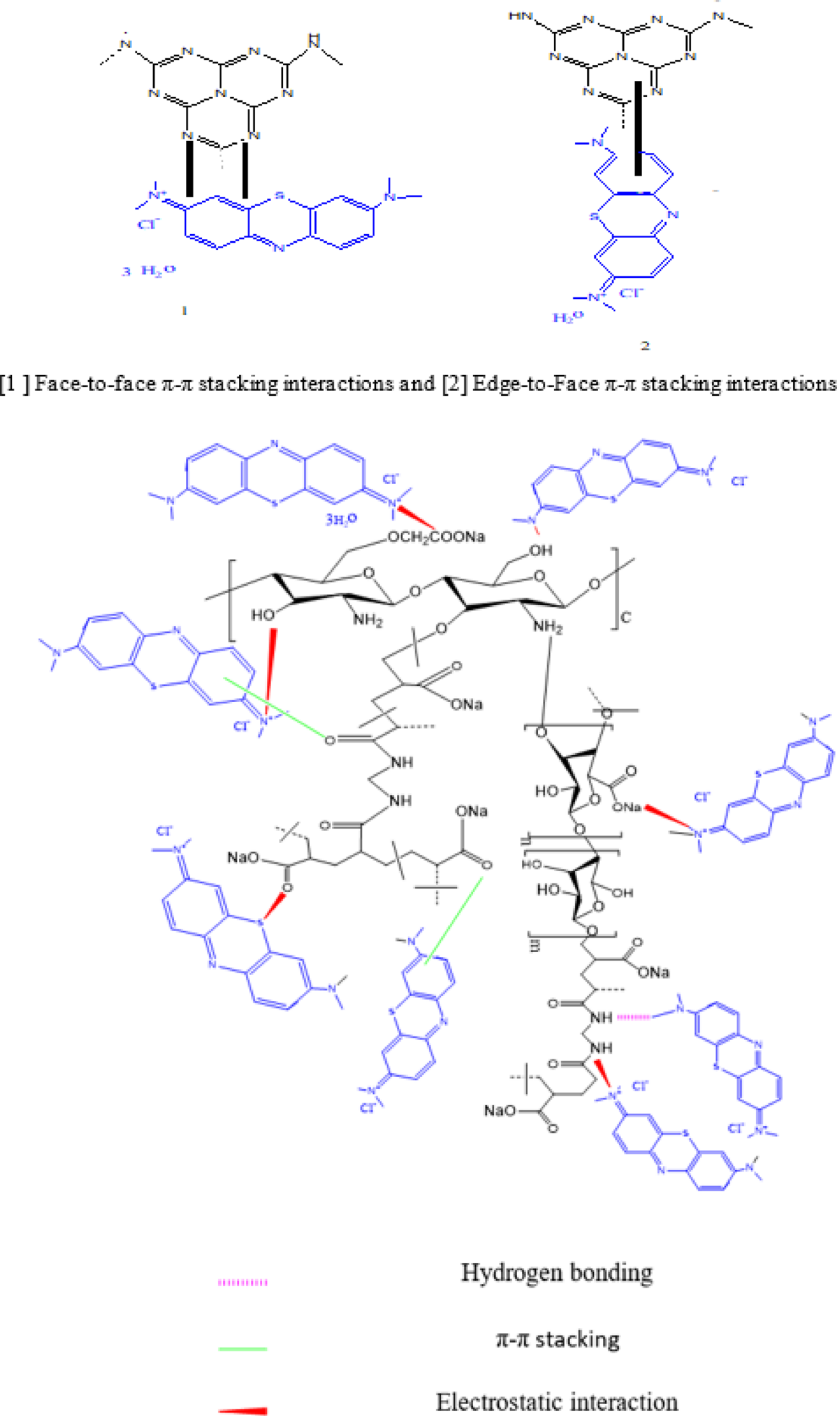
Proposed adsorption mechanism of MB dye by ZnO-g-C_3_N_4_@(CMCH/ALG)-g-PAA copolymer.

The practical adsorption data are well fitted by ZnO-g-C_3_N_4_(3%)@(CMCH/ALG)-g-PAA and (CMCH/ALG)-g-PAA adsorbents. This is evidenced by higher correlation coefficient *R*^2^ and the fitted *Q*_max_ (mg g^− 1^) values are close to practical data as shown in (Table 6). The constant *K*_L_ is calculated using the Langmuir model. *K*_L_ can be used to effectively evaluate whether the adsorption isotherm process is favorable (0 < *K*_L_ < 1) or unfavorable (*K*_L_ > 1). As seen in (Table 6) *K*_L_ is less than 1 for both ZnO-g-C_3_N_4_(3%)@(CMCH/ALG)-g-PAA and (CMCH/ALG)-g-PAA adsorbents implies that adsorption process is favorable (Scheme 3).

The Freundlich isotherm is an empirical model that assumes a heterogeneous adsorption, or a no uniform heat distribution on the adsorbent surface. The Freundlich equation is based on the assumption that the adsorption sites on the adsorbent surface are not evenly distributed, and adsorption is not constrained by single-layer adsorption, but the simultaneous occurrence of single-layer and multi-layer adsorption.

17 $$\:{\text{Q}}_{\text{e}}={\text{L}}_{\text{f}}\:{{\text{C}}_{\text{e}}}^{1/n}$$

*L*_f_ [ mg/g (L/mg)^n^ ] and *n* in the equation are the known as the Freundlich constants, where *n* gives the favorability of the adsorption technique and *L*_f_ is the adsorption capacity constant. If the value of *n* is equal to unity, the adsorption is linear; if the value is below to unity, this means the adsorption process is chemical; adsorption is a beneficial physical process if the value is greater than unity. As noticed in Fig. 9, the adsorption of MB dye with either ZnO-g-C_3_N_4_(3%)@(CMCH/ALG)-g-PAA and (CMCH/ALG)-g-PAA adsorbents is not consistent with Freundlich isotherm. The adsorption data not fitted with Freundlich equation.

The Temkin model proposes into account the effects of the interaction of the adsorbate and the adsorbing species. By ignoring the extremely low and large concentration values, the model assumes that the heat of adsorption (a function of temperature) of all of the molecules in the layer would decrease linearly rather than logarithmically with coverage due to adsorbate–adsorbent interactions.

18 $$\:{\text{Q}}_{\text{e}}=\frac{RT}{{\text{b}}_{\text{T}}}\left(ln{\text{C}}_{\text{e}}+ln\text{}{\text{A}}_{\text{T}}\right)$$

*A*_T_ is Temkin isotherm equilibrium binding constant (L g^− 1^), *b*_T_ (J mol^− 1^) is Temkin isotherm constant associated with adsorbate-adsorbent interactions, *R* is the universal gas constant (8.314 J/mol K^− 1^). The adsorption data of MB dye with ZnO-g-C_3_N_4_(3%)@(CMCH/ALG)-g-PAA and adsorbents are highly fitted with Temkin model this is evidenced by higher *R*^2^ as shown in (Table 6).

The Redlich–Peterson equation (Redlich and Peterson, 1959) is widely used as a compromise between Langmuir and Freundlich systems. This model has three parameters and incorporates the advantageous significance of both models. Redlich–Peterson model can be represented as follows:

19 $$Q_{e} = \frac{{k_{{RP~}} C_{e} }}{{1 + ~~\alpha _{{~R}} ~~C_{e} ^{{\beta _{{~R}} }} }}$$

*K*_*RP*_ (Lg^- 1^) and *α*_*R*_ (L mg^- 1^) are the Redlich–Peterson constants. *β*_*R*_ is exponent lies between 0 and 1. *C*_e_ is equilibrium liquid-phase concentration of the adsorbent (mg L^− 1^), and *Q*_e_ is equilibrium adsorbate loading on the adsorbent (mg g^−1^). At high liquid-phase concentrations of the adsorbate reduces to the Freundlich equation (Eq. 18):

20 $$\:{\text{Q}}_{\text{e}}=\frac{{{k}_{\text{RP}}\:{\text{C}}_{\text{e\:}}}^{1-\beta\:}}{{\alpha\:}_{\text{R}}}$$

Where, *k*_*RP*_ /*α*_*R*_ is Freundlich constant (*L*_f_) and (1-*β*_*R*_) = 1/*n* of the Freundlich isotherm model. When *β* = 1, reduces to Langmuir equation with *K*_L_ = *α*_*R*_ (Langmuir adsorption constant (L mg^−1^) which is related to the energy of adsorption and *k*_*RP*_ = Q_max_ is Langmuir maximum monolayer adsorption capacity of the adsorbent (mg g^−1^). When *β* = 0, reduces to Henry’s equation (Eq. 19) with *k*_HE_ representing Henry’s constant^82,83^.

21 $$\:{\text{Q}}_{\text{e}}=\:{\text{k}}_{\text{HE}}\:{\text{C}}_{\text{e}}$$

The Redlich-Peterson adsorption model was found to fit the experimental data for MB dye sufficiently in accordance with the higher correlation coefficients *R*^2^ = 0.9528 and 0.9599 for (CMCH/ALG)-g-PAA and ZnO-g-C_3_N_4_(3%)@(CMCH/ALG)-g-PAA, respectively. *β* equals 0.131 and 0.995 for (CMCH/ALG)-g-PAA and ZnO-g-C_3_N_4_(3%)@(CMCH/ALG)-g-PAA, respectively implying that adsorption process reduces to Langmuir equation as found in (Table 6).

Heterogeneous surfaces should be considerably better described by the Sips isotherm model which combines the Langmuir and Freundlich isotherm type models. The Sips isotherm approaches the Freundlich isotherm at low adsorbate concentrations, whereas at high concentrations it approaches the Langmuir isotherm.

22 $$\:{\text{Q}}_{\text{e}}=\frac{{{\text{Q}}_{\text{m}}\:\:\left({\text{k}}_{\text{s}}{\text{C}}_{\text{e\:}}\right)}^{1/n}}{{\:\:\left({\text{k}}_{\text{s}}{\text{C}}_{\text{e\:}}\right)}^{1/n}+1}$$

*Q*_m_ (mg/g) is the maximum adsorption capacity, and 1/*n* is the heterogeneity factor. *K*_S_ (L/mg) is the Sips equilibrium constants^84^. The Sips model is found to be applicable for fitting practical data of adsorption MB dye by (CMCH/ALG)-g-PAA (*R*^2^ = 0.9561) and ZnO-g-C_3_N_4_(3%)@(CMCH/ALG)-g-PAA (*R*^2^ = 0.9552).

**Table 6:** Adsorption models of MB dye by (CMCH/ALG)-g-PAA and ZnO-g-C_3_N_4_(3%)@(CMCH/ALG)-g-PAA.

Langmuir parameters				
	(CMCH/ALG)-g-PAA		ZnO-g-C_3_N_4_(3%)@(CMCH/ALG)-g-PAA	
*Q*_max_ (mg g^− 1^)	39.61 ± 22.9		34.59 ± 4.98	
*K*_L_(L mg^− 1^)	0.075 ± 0.06		0.2004 ± 0.65	
χ^2^	0.273		0.378	
*R* ^2^	0.9525		0.9552	

Freundlich				
	(CMCH/ALG)-g-PAA		ZnO-g-C_3_N_4_(3%)@(CMCH/ALG)-g-PAA	
*L*_f_	1.209		2.266	
*n*	0.165		0.094	
χ^2^	1.21		17.96	
*R* ^2^	0.9325		0.1729	

Temkin				
*RT*	1.75		1.33	
*A*_T_ (L g^− 1^)	3.43		0.159	
*b*_T_ (J mol^− 1^)	0.422		0.159	
*χ* ^2^	0.617		0.442	
*R* ^2^	0.8812		0.9651	

SIPS				
*Q*_m_ (mg g^− 1^)	31.40 ± 7.4		34.38 ± 2.0	
*K*_s_ (L mg^− 1^)	0.958 ± 0.95		4.32 ± 1.2	
*n*	8.77 ± 0.58		2.12 ± 5.8 × 10^− 26^	
*χ* ^2^	2.27		5.68	
*R* ^2^	0.9561		0.9552	

Redlich-Peterson				
*K*_RP_ (L g^− 1^)	1.28 ± 0.08		-3063.89 ± 1.7	
*α*_R_	3.24 ± 0.16		3087.98 ± 1.78	
*β*_R_	0.131 ± 0.06		0.9951 ± 0.28	
χ^2^	0.032		0.538	
*R* ^2^	0.9528		0.9599	

Fritz–Schlunder (F–S) isotherm				
	(CMCH/ALG)-g-PAA		ZnO-g-C_3_N_4_(3%)@(CMCH/ALG)-g-PAA	
*K*_FS_ (L mg^− 1^)	4.09 ± 0.66		22.53 ± 2.8	
*Q*_m_ (mg g^− 1^)	1.24 ± 0.22		5.74 ± 0.35	
*n*	0.39 ± 0.07		0.49 ± 0.15	
*χ* ^2^	0.680		11.7	
*R* ^2^	0.9736		0.8148	

The Fritz–Schlunder (F-S) model combines the Freundlich and Langmuir isotherms to form an empirical three-parameter isotherm (Eq. 21).

23 $$\:{\text{Q}}_{\text{e}}=\frac{{\text{k}}_{\text{FS\:}}\:{\text{Q}}_{\text{m}}\:\:{\text{C}}_{\text{e}}}{1+\:\:{\text{Q}}_{\text{m}}\:\:{\:{\text{C}}_{\text{e}}}^{\text{n}}}$$

When saturation is reached, *Q*_m_ is the amount of dye adsorbed (mg/g), *n* is the Freundlich constant, and *K*_FS_ is the Fritz–Schlunder model constant (L/mg).

Despite the higher correlation coefficient, the Fritz–Schlunder (F-S) model is not fitted with experimental adsorption data of MB dye by both (CMCH/ALG)-g-PAA and ZnO-g-C_3_N_4_(3%)@(CMCH/ALG)-g-PAA. The fitted maximum adsorption capacity *Q*_m_ (mg g^− 1^) values are not consistent with experimental data as seen in (Table 6).

#### Adsorption thermodynamics

**Fig. 10:**
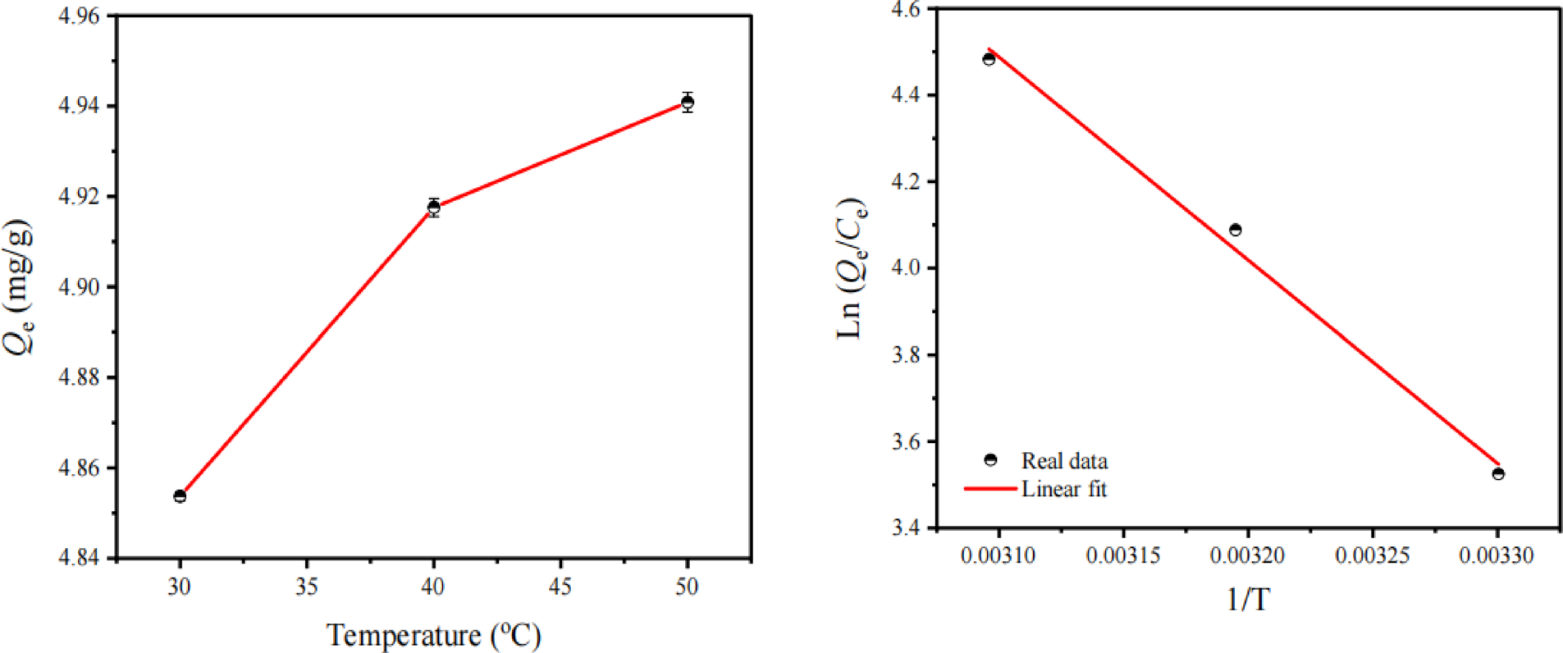
Adsorption dynamic of MB dye by ZnO-g-C_3_N_4_(3%)@(CMCH/ALG)-g-PAA.

The adsorption thermodynamic parameters of ZnO-g-C_3_N_4_(3%)@(CMCH/ALG)-g-PAA nanocomposite are summarized in (Table 7 and Fig. 10). Δ*H*° is positive for ZnO-g-C_3_N_4_(3%)@(CMCH/ALG)-g-PAA nanocomposite which indicates the adsorption process is endothermic, whereas the positive Δ*S*° values indicate randomness during MB adsorption^85^. Furthermore, the values of Δ*H*◦ are positive and less than 42 kJ/mol, which also suggests that the adsorption process in this study is physisorption^86^. The positive value of Δ*S*^0^ reflects the affinity between MB and of ZnO-g-C_3_N_4_(3%)@(CMCH/ALG)-g-PAA nanocomposite^87^. The larger Δ*S*° values implies larger degrees of freedom at the solid–liquid interface during adsorption which causes structural changes to the adsorbent and can influence the rate of adsorption^85^. The Δ*G* decreases with increasing temperature suggests that higher temperature is good for the adsorption process and all the heat absorbed by the system is used to increase enthalpy^50^.

The Δ*G*◦ values in all adsorption experiments are larger than − 20 kJ/mol, which indicates that the adsorption is physisorption^88^. According to Hamid Safarzadeh et al. (2023), sodium alginate-grafted-poly(methacrylic acid-co-acrylamide)/montmorillonite (SA-g-poly(MMA-co-AAm)/MMT) nanocomposite hydrogel was successfully fabricated (Table 8. The outcomes showed that it was prepared successfully. At 25 °C, a pH of 7, a dosage of 1.5 g/L, a contact period of 90 min, an MG concentration of 10 mg/L, and a pH of 7, the adsorbent’s maximum adsorption capacity of 95.91 mg/g was achieved. A negative Gibbs free energy (ΔG°) value in thermodynamics tests revealed that the adsorption process was spontaneous. Furthermore, the adsorption enthalpies (ΔH°) of SA-g-poly(MMA-co-AAm) and SA-g-poly(MMA-co-AAm)/MMT nanocomposite hydrogels were − 61.512 and − 77.281 kJ/mol, respectively, demonstrating the exothermic nature of the MG adsorption when employing these adsorbents^76^.

**Table 7:** Adsorption thermodynamic parameters.

*R* ^2^	RSS	Temp (K)	$\:{\varDelta\:G}^{O}$(kJ/mol)	ΔH° (kJ/mol)	$\:{\varDelta\:S}^{O}$(J/mol)
0.9930	0.0032	303	-8.88	39.02	158.27
		313	-10.63		
		323	-12.03		

**Table 8:** Comparison with other adsorbent: maximum adsorption capacity and adsorption kinetics.

Composite adsorbents	Max. adsorption capacity (mg/g)	Adsorption conditions	Adsorption kinetics	Ref.
ZnO-g-C_3_N_4_@(CMCH/ALG-g-PAA)	24.30	MB:25 ppm, 0.1 L, 145 min, pH 8.0, 0.1 g	*Pseudo first order*: 0.014 min^− 1^ *Elovich*: α: 0.482 mg g^− 1^ min^− 1^ β: 0.062 g min^− 1^	This work
Xylan and gelatin-based hydrogel	26.04	120 min, pH 5.84	*Pseudo second order* : 0.182 g mg^− 1^ min^− 1^	^89^
Carboxymethyl cellulose grafted polyacrylamide/carbon black nanocomposite hydrogel	27.32	pH 7, 30 min, dose 1.5 g/L, Initial concentration :10 mg/L	*Pseudo second order*: 0.0321 g mg^− 1^ min^− 1^ *Elovich*: α: 127.6 mg g^− 1^ min^− 1^ β: 0.612 g min^− 1^	^90^
Gum acacia capped polyaniline-based nanocomposite hydrogel	35.41	pH 10 , Initial concentration: 10 mg L^− 1^	*Pseudo second order*: 0.523 g mg^− 1^ min^− 1^	^91^
Corn starch, phosphate corn starch, starch nanocrystals and phosphate corn starch nanocrystals	88.53	pH 9, 45 °C, 15 min Initial concentration: 500 mg L^−^ ^1^	*Pseudo second order*: 0.695 g mg^− 1^ min^− 1^	^92^
Cellulose–Polyvinyl alcohol supported magnetic nanocomposites from lentil husk	46.51	120 min, pH8	*Pseudo second order*: 0.020 g mg^− 1^ min^− 1^ *Elovich*: α: 41.11 mg g^− 1^ min^− 1^ β: 1.12 g min^− 1^	^93^

## Conclusion

The developed multiple functional (carboxymethyl chitosan/alginate)-grafted polyacrylic acid composite (CMCH/ALG)-g-PAA with graphitic carbon nitride (ZnO-g-C_3_N_4_) as a high-potential filler has succeeded in meeting the growing demand for high-performance advanced polymer composites for highly effective dye removal from wastewater. At pH 8.0, pH 5.8, pH 7, and saline solution, the maximum swelling ratios were 1697.10% 1496.24%, 775.99%, and 140.5%, respectively. The swelling capacity data was well-fitted by the Vigot and first order models. The greatest adsorption capacity for (CMCH/ALG)-g-PAA with 3% ZnO-g-C_3_N_4_ was 24.30 mg/g at adsorbate concentration (0.1 g/L), MB dye concentration (25 mg/L), and pH 8.0. The Langmuir, Temkin, Redlich-Peterson, and Sips models are able to suit the practical data of adsorption indicated that adsorption mechanism is a complicated process. Adsorption is physisorption since all adsorption investigations had Δ*G*◦ values greater than − 20 kJ/mol. Adsorption is physisorption since all adsorption investigations had Δ*G*◦ values greater than − 20 kJ/mol. This study offers an intriguing, useful, and environmentally friendly method for efficiently eliminating cationic dyes from wastewater.

## Data Availability

The datasets generated during and/or analyzed during the current study are available from the corresponding author on reasonable request.
